# A maternal high-fat diet induces fetal origins of NASH-HCC in mice

**DOI:** 10.1038/s41598-022-17501-8

**Published:** 2022-07-30

**Authors:** Takao Takiyama, Toshihiro Sera, Masanori Nakamura, Masato Hoshino, Kentaro Uesugi, Shin-ichi Horike, Makiko Meguro-Horike, Ryoichi Bessho, Yuri Takiyama, Hiroya Kitsunai, Yasutaka Takeda, Kazuki Sawamoto, Naoto Yagi, Yuji Nishikawa, Yumi Takiyama

**Affiliations:** 1 Division of Diabetes, Department of Medicine, Asahikawa Medical University, Asahikawa, Japan; 2 Department of Mechanical Engineering, Faculty of Engineering, Kyushu University, Fukuoka, Japan; 3 Department of Electrical and Mechanical Engineering, Nagoya Institute of Technology, Nagoya, Japan; 4 Japan Synchrotron Radiation Research Institute, Sayo-cho, Japan; 5 Advanced Science Research Center, Kanazawa University, Kanazawa, Japan; 6 Department of Pathology, Asahikawa Medical University, Asahikawa, Japan

**Keywords:** Cancer, Endocrinology, Gastroenterology

## Abstract

Maternal overnutrition affects offspring susceptibility to nonalcoholic steatohepatitis (NASH). Male offspring from high-fat diet (HFD)-fed dams developed a severe form of NASH, leading to highly vascular tumor formation. The cancer/testis antigen HORMA domain containing protein 1 (HORMAD1), one of 146 upregulated differentially expressed genes in fetal livers from HFD-fed dams, was overexpressed with hypoxia-inducible factor 1 alpha (HIF-1alpha) in hepatoblasts and in NASH-based hepatocellular carcinoma (HCC) in offspring from HFD-fed dams at 15 weeks old. Hypoxia substantially increased *Hormad1* expression in primary mouse hepatocytes. Despite the presence of three putative hypoxia response elements within the mouse *Hormad1* gene, the *Hif-1alpha* siRNA only slightly decreased hypoxia-induced *Hormad1* mRNA expression. In contrast, N-acetylcysteine, but not rotenone, inhibited hypoxia-induced *Hormad1* expression, indicating its dependency on nonmitochondrial reactive oxygen species production. Synchrotron-based phase-contrast micro-CT of the fetuses from HFD-fed dams showed significant enlargement of the liver accompanied by a consistent size of the umbilical vein, which may cause hypoxia in the fetal liver. Based on these findings, a maternal HFD induces fetal origins of NASH/HCC via hypoxia, and HORMAD1 is a potential therapeutic target for NASH/HCC.

## Introduction

The prenatal environment is a nongenetic factor that leads to epigenetic changes in early development^1^. Support for this pathogenetic mechanism comes from epidemiological studies of low-birth-weight infants during wartime^2^. Currently, 50% of women of reproductive age are obese^3^, and one in seven women has gestational diabetes at birth^4^, which is affected by maternal hyperglycemia. Fetal exposure to maternal diabetes and hyperglycemia may contribute considerably to the global diabetes epidemic. A previous cohort study reported increased erythropoietin levels in cord blood with an increase in the maternal body mass index without differences in placental lesions, likely as a result of chronic fetal hypoxia^5^. Fetal hypoxia has also been suggested to cause increased erythropoiesis on the first day after birth in infants of mothers with diabetes^6^. In addition, maternal obesity is associated with placental hypoxia and may increase the risk of severe fetal and neonatal complications^7^. Therefore, studies examining the metabolic abnormalities that overnutrition during fetal life may cause in adulthood are urgently needed.

Nonalcoholic fatty liver disease (NAFLD) is the most prevalent chronic liver disease^8^. NAFLD affects 70–80% of patients with type 2 diabetes and 30–40% of adults with type 1 diabetes^9,10^. Currently, one in four members of the adult population worldwide is affected by NAFLD. NASH, the most advanced form of NAFLD, has a high risk of progressing to cirrhosis and hepatocellular carcinoma and is becoming a major cause of end-stage liver disease. In particular, approximately half of all NAFLD cases in children have progressed to NASH by the time of diagnosis^11^. Because 20% of patients with NASH will develop cirrhosis, NASH will become the leading indication for liver transplantation in the US^12^. Actually, the incidence of liver transplantations for NASH cirrhosis in individuals aged 18 to 40 years in the US increased from 0.53% in 2002 to 4.46% in 2012, indicating an annual increase rate of 14% per year in liver transplant indications for NASH^13^. The younger age of onset of NASH suggests an increasing effect of the prenatal nutritional environment. A Swedish nationwide study indicated that maternal obesity increased the risk and severity of NAFLD in offspring^14^.

In this study, we investigated the effect of prenatal overnutrition on the fetal liver and subsequent development of NAFLD/NASH/HCC in offspring to clarify the mechanism and identify a new target for NASH/HCC.

## Results

### A maternal high-fat diet induces NASH in offspring

**Figure 1:**
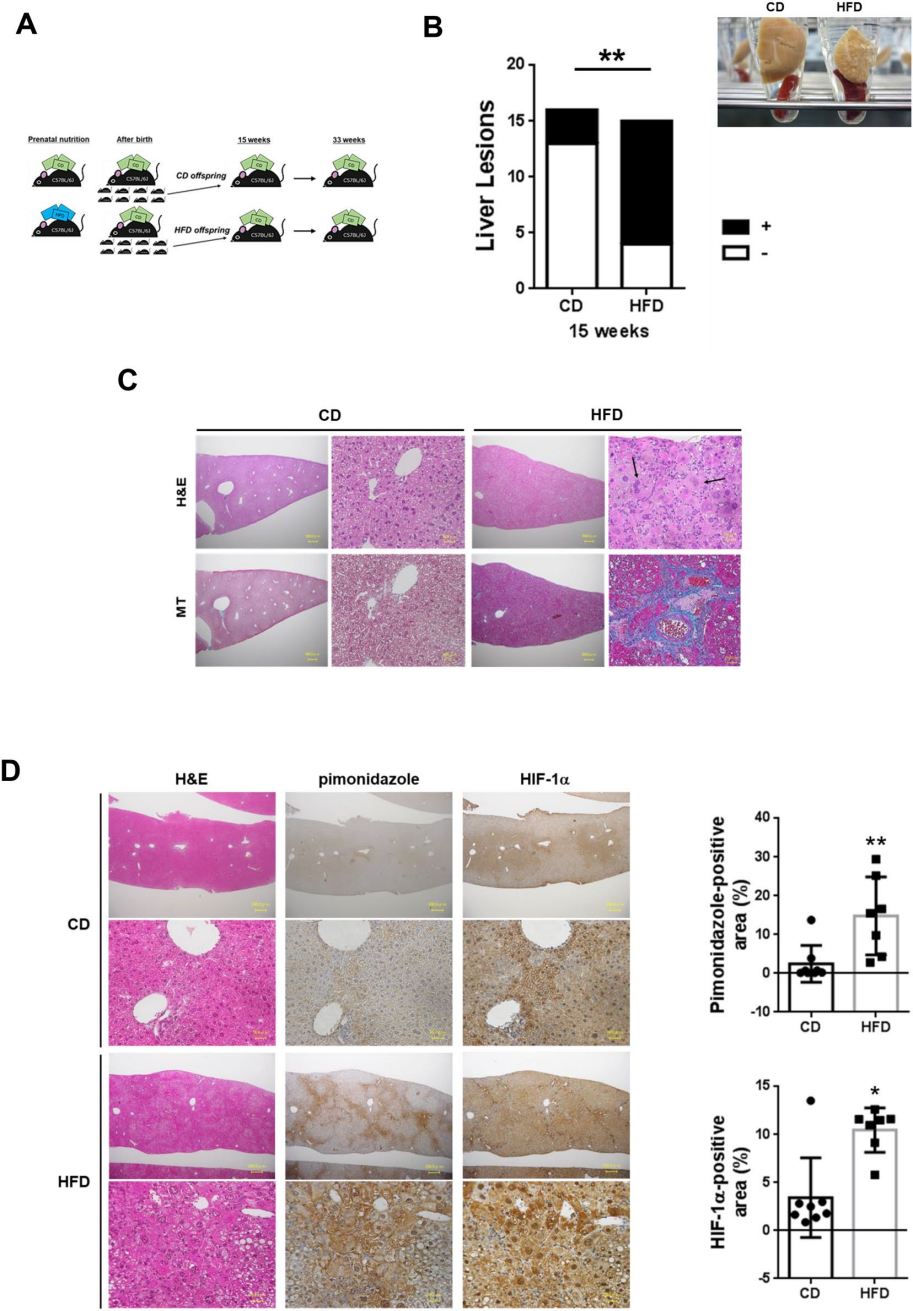

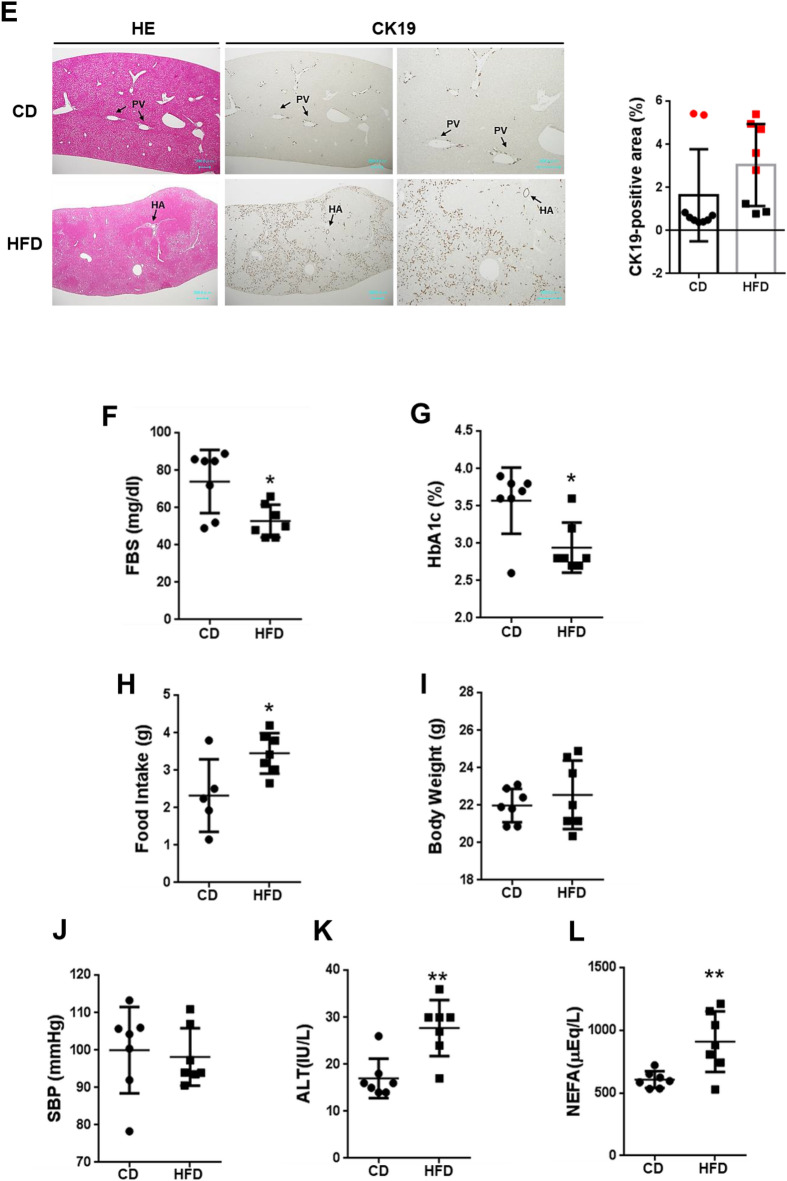
Maternal HFD consumption induces hepatic steatosis in offspring. (**A**) Experimental setup and time points that were analyzed. Female C57BL/6J mice were fed either a HFD or a CD during gestation. During lactation and after weaning, all mice were fed a CD. (**B**) The number of 15-week-old offspring with liver lesions indicating steatohepatitis (steatosis, lobular inflammation, hepatocellular ballooning, and fibrosis) in the CD (n = 16) and HFD groups (n = 15). Photographs of liver sections and spleens from 15-week-old offspring of the CD- and HFD-fed dams. Statistical analysis: Fisher’s exact test. (**C**) Hematoxylin and eosin staining (upper panels) and Masson’s trichrome (MT) staining (lower panels) of the livers from offspring of HFD-fed dams. The atypical cells exhibited severe dysplasia, with an increased nuclear-cytoplasmic ratio and enlarged nuclei with hyperploidization (black arrows) accompanied by fat deposition. Scale bars: 300 μm (low magnification) and 30 μm (high magnification). (**D**) The liver sections from offspring of CD- and HFD-fed dams were stained for pimonidazole and HIF-1α. Representative images of pimonidazole and HIF-1α staining (cells stained brown) are shown. Scale bars: 300 μm (low magnification) and 30 μm (high magnification) in each group. Quantification of the pimonidazole-positive area and HIF-1α-positive area (CD = 8 offspring from 3 dams, HFD = 7 offspring from 3 dams). *p < 0.05 and **p < 0.01 using the Mann–Whitney test. (**E**) CK19 immunohistochemistry in the liver sections from offspring of CD- and HFD-fed dams. Scale bars: 300 μm. *PV* portal vein, *HA* hepatic artery. Quantification of the CK19-positive area. *p* > 0.05 using the Mann–Whitney test (CD = 9 offspring from 3 dams, HFD = 8 offspring from 3 dams). Two red circles and five red rectangles show the results for offspring with liver lesions. The stained sections were observed and visualized using a light microscope system (BZ-8100; Keyence, Osaka, Japan. https://www.keyence.com). (**F**) FBS, (**G**) HbA1c, (**H**) food intake/day, (**I**) body weight, (**J**) SBP, (**K**) ALT, and (**L**) NEFA levels. The values represent individual measurements and are presented as the means ± SD. The significance of the differences between groups was determined using unpaired Student’s *t* tests. Welch’s corrections were used when the variances between groups were unequal. *p < 0.05 and **p < 0.01.

We generated a novel mouse model to examine the effect of intrauterine overnutrition on the liver pathophysiology in offspring. Female C57BL/6J mice at 8 weeks of age were fed a high-fat diet (HFD) for only the gestational period, which was different from previous studies using animal models of maternal HFD exposure and examining the effects on metabolism in offspring^15^ (Fig. 1A). We observed no difference in the weekly body weight of male offspring from control diet (CD)-fed dams and HFD-fed dams (Supplementary Fig. 1). Importantly, 15-week-old male offspring from HFD-fed dams had a severe form of NASH accompanied by splenomegaly (Fig. 1B). Maternal HFD consumption significantly induced the formation of liver lesions in 15-week-old offspring (*p* < 0.01, Fig. 1B). Liver sections from offspring of HFD-fed dams showed lobular inflammation (Fig. 1C). The atypical cells exhibited severe dysplasia, with an increased nuclear-cytoplasmic ratio and enlarged nuclei with hyperploidization^16,17^ accompanied by fat deposition, ductular reaction^18^ and fibrosis in zone 3 (the centrilobular region), as evaluated using Masson’s trichrome staining (blue staining, Fig. 1C). These findings were strikingly different from the pathological findings of livers from other HFD-fed NASH mouse models reported previously^15^. C57BL/6J mice have mutations in the nicotinamide nucleotide transhydrogenase (*Nnt*) gene and glucokinase (*Gck*)^19^. Energy metabolism influences the contribution of NNT to NADPH-dependent mitochondrial peroxide metabolism in the liver^20^. We examined the effect of a maternal HFD using C57BL/6N mice, which have no mutation of the *Nnt* gene, to determine the effect of the *Nnt* mutation on C57BL/6J mice^19^. The offspring of HFD-fed C57BL/6N dams also showed NASH in the livers, which was observed in C57BL/6J mice (Supplementary Fig. 2). We did not observe a sex difference in maternal HFD-induced liver injury (Supplementary Fig. 3).

The offspring of CD-fed dams showed weak positive staining for both pimonidazole and hypoxia-inducible factor 1 alpha (HIF-1α) in zone 3 (Fig. 1D), which borders the central vein and is associated with detoxification, aerobic metabolism, glycolysis and hydrolysis. Perivenous zone 3 presents a lower oxygen concentration (30–35 mmHg) than periportal zone 1 (60–65 mmHg)^21^, which borders the portal tracts and functions in hepatocyte regeneration, bile duct proliferation and gluconeogenesis. Consistent with the oxygen distribution, the offspring of CD-fed dams without liver lesions were negative for pimonidazole and HIF-1α staining in zone 1 (Fig. 1D). The livers of offspring from HFD-fed dams exhibited very strong pimonidazole (*p* < 0.01, CD vs. HFD. Figure 1D) and HIF-1α staining in large atypical cells in zone 3 (*p* < 0.05, CD vs. HFD. Fig. 1D). Accordingly, the number of cytokeratin 19 (CK19)-positive bile ductules was increased in zone 3 in the liver sections from offspring of HFD-fed dams, but a significant difference in the CK-positive areas was not observed between the two groups of offspring from CD-fed dams with steatohepatitis (red circles in the right graph in Fig. 1E). Although zone 1 in the livers of offspring from HFD-fed dams showed arterial dilation, arterial perfusion did not restore hypoxia in fibrotic zone 3 lesions, suggesting that hemodynamic changes promoted an oxygen redistribution in the liver.

Moreover, compared with the offspring from CD-fed dams, the offspring from HFD-fed dams showed a lower fasting blood sugar (FBS) level (74.00 ± 16.95 vs. 52.86 ± 8.71 mg/dL, *p* < 0.05, Fig. 1F) and HbA1c level (3.57 ± 0.44 vs*.* 2.94 ± 0.34%, *p* < 0.05, Fig. 1G), despite greater food intake (2.33 ± 0.97 vs. 3.45 ± 0.54 g/day, *p* < 0.05, Fig. 1H). Body weight (21.98 ± 0.90 vs. 22.55 ± 1.82 g, Fig. 1I) and systolic blood pressure (SBP) (100 ± 11.54 vs. 98.19 ± 7.7 mmHg, Fig. 1J) were not different between the groups at 15 weeks of age. Remarkably, levels of alanine aminotransferase (ALT) (17.00 ± 4.2 vs. 27.71 ± 5.95 IU/L, *p* < 0.01, Fig. 1K) and plasma nonesterified fatty acids (NEFAs) (608.0 ± 66.61 vs. 911.0 ± 242.4 microEq/L, *p* < 0.01, Fig. 1L) were increased in the offspring from HFD-fed dams. These changes were consistent with the histological changes associated with NASH and cirrhosis (Supplementary Table 1, Fig. 1B–E).

### HORMA domain containing protein 1 (*Hormad1*) is upregulated in the liver of fetuses from HFD-fed dams

**Figure 2:**
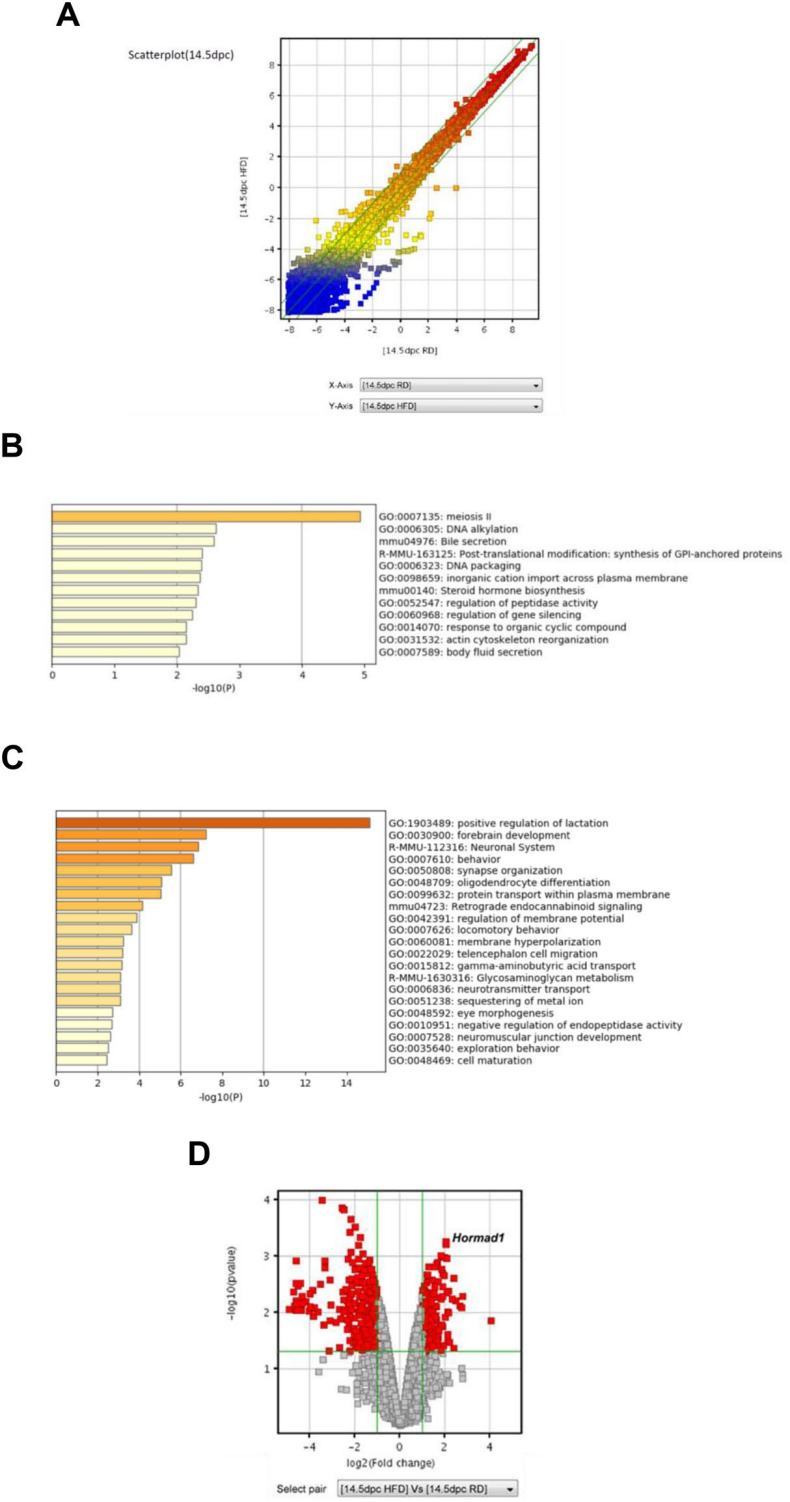
Maternal HFD consumption upregulates *Hormad1* gene expression in the fetal liver. (**A**) Scatterplot of the gene expression profiles for hepatocytes from fetuses (14.5 dpc). Green lines indicate the cutoffs for twofold up- and downregulation. Enrichment scores obtained from the Gene Ontology enrichment analysis of the selected mRNAs. (**B**) GO analysis of the upregulated genes in livers from fetuses of HFD-fed dams compared with CD-fed dams. (**C**) GO analysis of the downregulated genes in livers from fetuses of HFD-fed dams compared with CD-fed dams. (**D**) Volcano plots of the gene expression data. The horizontal axis represents the log2 (fold change), and the vertical axis represents the  − log 10 (p value). The red plots represent the selected DEGs. Volcano plot showing *Hormad1,* which had the lowest *p* value (*p* = 0.0006), and a greater than four-fold change (log2 = 2.037) in expression in livers from fetuses of HFD-fed dams compared with CD-fed dams. Data are presented using GeneSpring software 12.1 (Agilent Technologies. https://www.agilent.com).

HFD consumption during gestation induced maternal obesity at E14.5, as evidenced by the 40% increase in body weight (11.81 ± 0.20 vs*.* 8.44 ± 0.4 g, HFD vs*.* CD, *p* = 0.001) without significant difference in blood sugar levels (108.50 ± 23.33 vs*.* 153.00 ± 10.00 mg/dL, HFD vs*.* CD, *p* > 0.05) as reported in the previous study^22^. We assayed mRNA levels with a microarray (SuperPrint G3 Mouse GE 8 × 60 K, Agilent, Santa Clara, CA, USA) to identify genes and pathways targeted by the maternal HFD in fetal livers (Fig. 2A). We identified 430 differentially expressed genes (DEGs), including 146 upregulated and 284 downregulated genes, in the livers of fetuses from HFD-fed dams compared with those from CD-fed dams (Fig. 2A, Table 1). The upregulated DEGs were significantly enriched in meiosis II, DNA alkylation, and bile secretion (Fig. 2B), and the downregulated DEGs were mainly enriched in the positive regulation of the lactation neuronal system and cell maturation (Fig. 2C). Notably, among the upregulated DEGs, we found that the cancer/testis (CT) antigen^23,24^ *Hormad1* was present at four-fold higher in fetal livers from HFD-fed dams than in those from CD-fed dams and showed the greatest statistically significant difference (fold change log2 = 2.037, *p* = 0.0006) (Fig. 2D, Table 1). *Hormad1*, also called *Nohma*, has been identified to be transcribed in human^25^ and mouse testes and ovaries^26^. CT antigens are normally expressed in the germline but are ectopically expressed in various cancers^24^ (Supplementary Table 2), including gastric cancer^27^, triple-negative breast cancers^28,29^, lung cancer^30,31^ and ovarian cancer^32,33^, indicating that they may be potential biomarkers and therapeutic targets for cancers^34^, such as hepatocellular carcinoma^35^.

**Table 1:** Differentially expressed genes in fetal livers of offspring of HFD-fed dams vs*.* CD-fed dams.

Genbank accession	Gene symbols	Gene name	log2 Fold change
**Up-regulated differentially expressed genes (DEGs) in fetal livers of HFD-fed dams**			
1NM_181549	Clec18a	C-type lectin domain family 18, member A	4.0055
2NM_010021	Dazl	Deleted in azoospermia-like	2.7656
3NM_009978	Cst8	Cystatin 8 (cystatin-related epididymal spermatogenic)	2.7266
4NM_175296	Mael	Maelstrom homolog (Drosophila)	2.6353
5NM_009978	Cst8	Cystatin 8 (cystatin-related epididymal spermatogenic)	2.4424
6NM_011472	Sprr2f	Small proline-rich protein 2F	2.3800
7NM_001007576	Gucy2f	Guanylate cyclase 2f	2.1754
8NM_177350	Gldn	Gliomedin	2.1552
9NM_027582	Akr1cl	Aldo–keto reductase family 1, member C-like	2.1217
10NM_011089	Pira2	Paired-Ig-like receptor A2	2.1095
11NM_007663	Cdh16	Cadherin 16	2.0972
12NM_001167705	Serpina3a	Serine (or cysteine) peptidase inhibitor, clade A, member 3A	2.0852
13NM_001277891	Fkbp6	FK506 binding protein 6	2.0789
14NM_001289534	Hormad1	HORMA domain containing 1	2.0367
15NM_001166433	AU022751	Expressed sequence AU022751	1.9846
16NM_011376	Sim1	Single-minded homolog 1 (Drosophila)	1.9375
17NM_028610	Dppa4	Developmental pluripotency associated 4	1.8945
18NM_019779	Cyp11a1	Cytochrome P450, family 11, subfamily a, polypeptide 1	1.8736
19NM_181549	Clec18a	C-type lectin domain family 18, member A	1.8421
20BC015294	Glyat	Glycine-*N*-acyltransferase	1.8186
**Down-regulated differentially expressed genes (DEGs) in fetal livers of HFD-fed dams**			
1NM_026087	Ceacam12	Carcinoembryonic antigen-related cell adhesion molecule 12	− 4.9153
2NM_023741	Prl8a8	Prolactin family 8, subfamily a, member 81	− 4.7050
3NM_011963	Psg18	Pregnancy specific glycoprotein 18	− 4.6818
4NM_025957	Ceacam14	Carcinoembryonic antigen-related cell adhesion molecule 14	− 4.6157
5NM_023289	Ceacam11	Carcinoembryonic antigen-related cell adhesion molecule 11	− 4.6113
6NM_026906	Cts3	Cathepsin 3	− 4.6024
7NM_001271378	Prl8a6	Prolactin family 8, subfamily a, member 6	− 4.5822
8NM_011168	Prl7a2	Prolactin family 7, subfamily a, member 2	− 4.5645
9NM_011963	Psg18	Pregnancy specific glycoprotein 18	− 4.5130
10NM_023332	Prl8a9	Prolactin family8, subfamily a, member 9	− 4.4265
11NM_001037168	Psg27	Pregnancy-specific glycoprotein 27	− 4.4117
12NR_002857	Psg-ps1	Pregnancy specific glycoprotein pseudogene 1	− 4.3844
13NM_025532	Prl2b1	Prolactin family 2, subfamily b, member 1	− 4.3217
14NM_028480	Ceacam5	Carcinoembryonic antigen-related cell adhesion molecule 5	− 4.3213
15NM_001029893	Psg26	Pregnancy-specific glycoprotein 26	− 4.1893
16NM_007676	Psg16	Pregnancy specific glycoprotein 16	− 4.0256
17NM_027403	Psg21	Pregnancy-specific glycoprotein 21	− 4.0000
18NM_026429	Tpbpb	Trophoblast specific protein beta	− 3.9700
19NM_009411	Tpbpa	Trophoblast specific protein alpha	− 3.9107
20NM_027210	Ceacam13	Carcinoembryonic antigen-related cell adhesion molecule 13	− 3.8535

### Maternal HFD consumption induces HORMAD1 protein expression in the livers of fetuses and adult offspring

**Figure 3:**
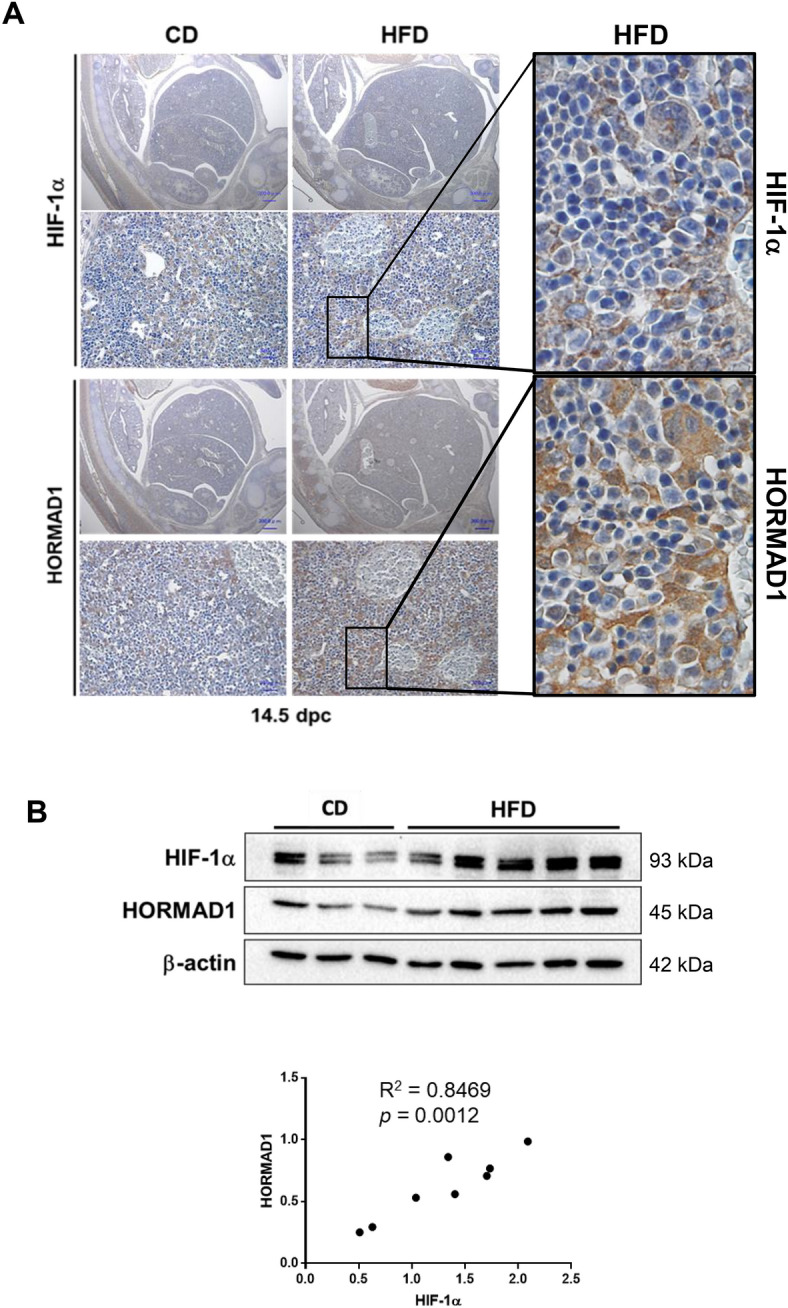

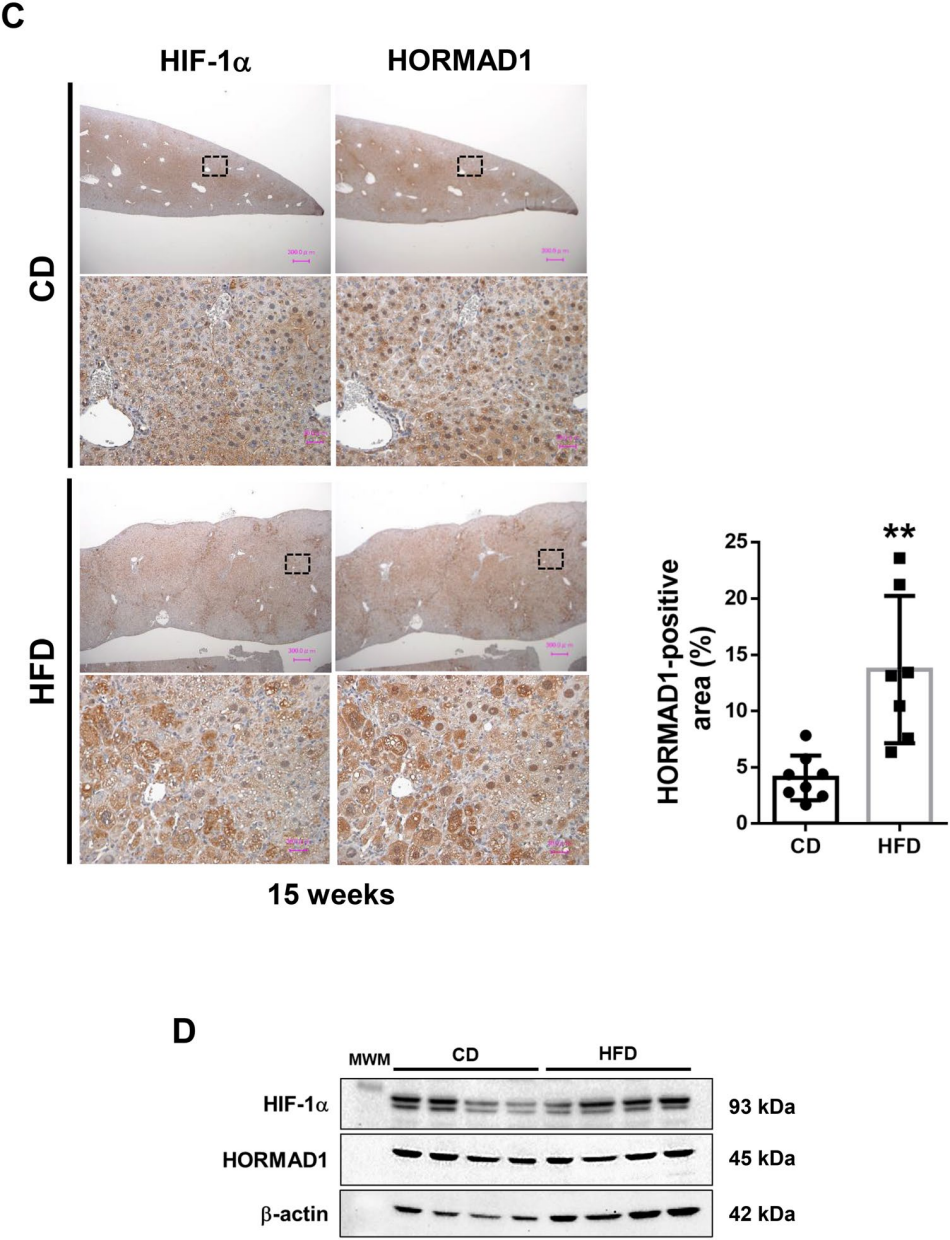

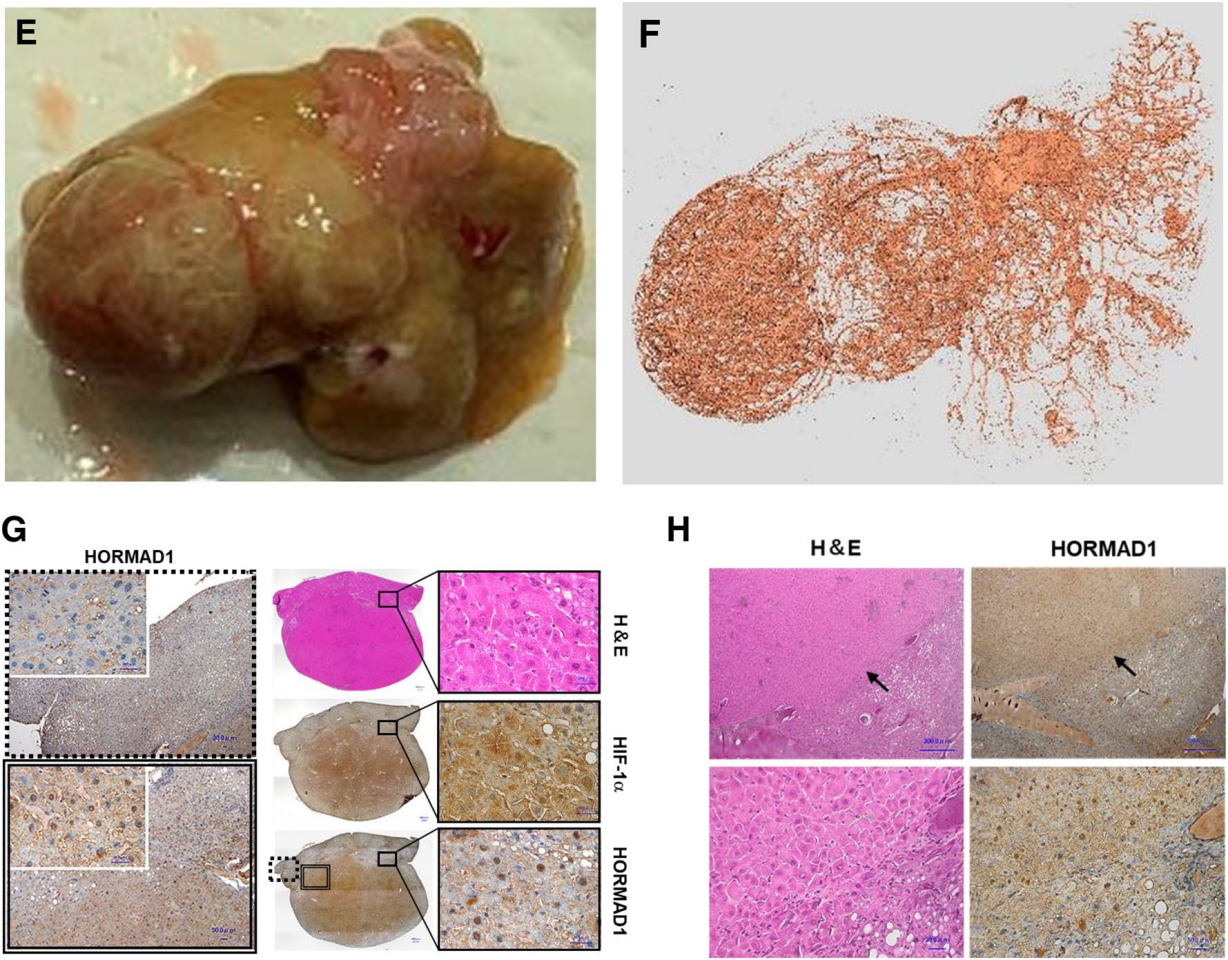
HORMAD1 colocalizes with HIF-1α in fetal livers. (**A**) Representative photomicrographs of immunohistochemical staining for HIF-1α and HORMAD1 in fetal livers at 14.5 dpc. Scale bars: 300 μm (low magnification) and 30 μm (high magnification). Insets show the magnified images from the left panels. (**B**) Western blot analysis showed positive correlations between HORMAD1 and HIF-1α levels in the fetal livers of mice (R^2^ = 0.8469, *p* = 0.0012). (**C**) Immunohistochemical staining for HIF-1α and HORMAD1 in the livers of offspring at 15 weeks of age. Scale bars: 300 μm (low magnification) and 30 μm (high magnification). Quantification of HORMAD1-positive area (CD = 8 offspring from 3 dams, HFD = 7 offspring from 3 dams). **p < 0.01 using the Mann–Whitney test. (**D**) Western blot analysis of HIF-1α and HORMAD1 levels in the livers of 15-week-old offspring. MWM: 117 kDa band in the molecular weight marker (HiMark Prestained protein standard, Thermo Fisher Scientific). (**E**) Macroscopic image of the liver after barium perfusion of offspring from HFD-fed dams at 33 weeks old. (**F**) Visualization of 3D vessels in the liver of offspring from HFD-fed dams at 33 weeks old using synchrotron micro-CT and Amira software (version 1.4.0, https://www.thermofisher.com). (**G**) Immunohistochemical staining for HIF-1α and HORMAD1 in hepatic tumors from 33-week-old offspring of HFD-fed dams. Insets in the middle panels show the magnified images (right panels). HORMAD1 expression in peritumoral lesions (dotted line) and intratumoral lesions (double lines) in hepatic tumors from 33-week-old offspring of HFD-fed dams. Insets in the left panels show the magnified image of each lesion. (**H**) Capsular invasion of a hepatic tumor. Arrows show the area of invasion. Bars: 300 μm (low magnification) and 30 μm (high magnification).

Then, we examined HORMAD1 protein expression in the livers of fetuses and adult offspring using immunohistochemistry and western blotting. Immunohistochemical staining showed that HORMAD1 protein expression was upregulated in hepatoblasts in fetal livers and colocalized with HIF-1α (Fig. 3A). In addition, the western blot analysis revealed that HORMAD1 protein expression was positively associated with HIF-1α protein expression in fetal livers (R^2^ = 0.8469, *p* = 0.0012, Fig. 3B).

On the other hand, the expression levels of the HORMAD1 and HIF-1α proteins were increased in NASH lesions of 15-week-old offspring from HFD-fed dams (Fig. 3C). NASH includes the histological spectrum of lesions ranging from steatosis to a complex pattern characterized by hepatocellular injury and inflammation^36^. Because the proteins were extracted from various lesions in the livers of 15-week-old offspring from HFD-fed dams, western blot analysis might fail to identify a significant correlation between HORMAD1 and HIF-1α protein expression (Fig. 3D).

Usually, offspring of HFD-fed dams die because of severe cirrhosis, not cancer. However, we found that some offspring of HFD-fed dams developed HCC without severe cirrhosis. We subsequently evaluated HORMAD1 expression in hepatic tumors of 33-week-old offspring from HFD-fed dams to clarify the malignant potential of this mouse model and the role of HORMAD1 in hepatocellular carcinogenesis (Fig. 3E), and the results showed abundant tumor vascularization in synchrotron micro-CT scans (Fig. 3F), which is observed in humans with HCC^37^. Both HIF-1α and HORMAD1 showed nuclear localization in hepatic tumors. However, peritumoral lesions did not express HORMAD1 (Fig. 3G). Hepatic tumors also presented capsular invasion, indicating malignant features such as those associated with hepatocellular carcinoma (Fig. 3H).

### Hypoxia induces HORMAD1 expression in mouse primary hepatocytes (MPHs)

**Figure 4:**
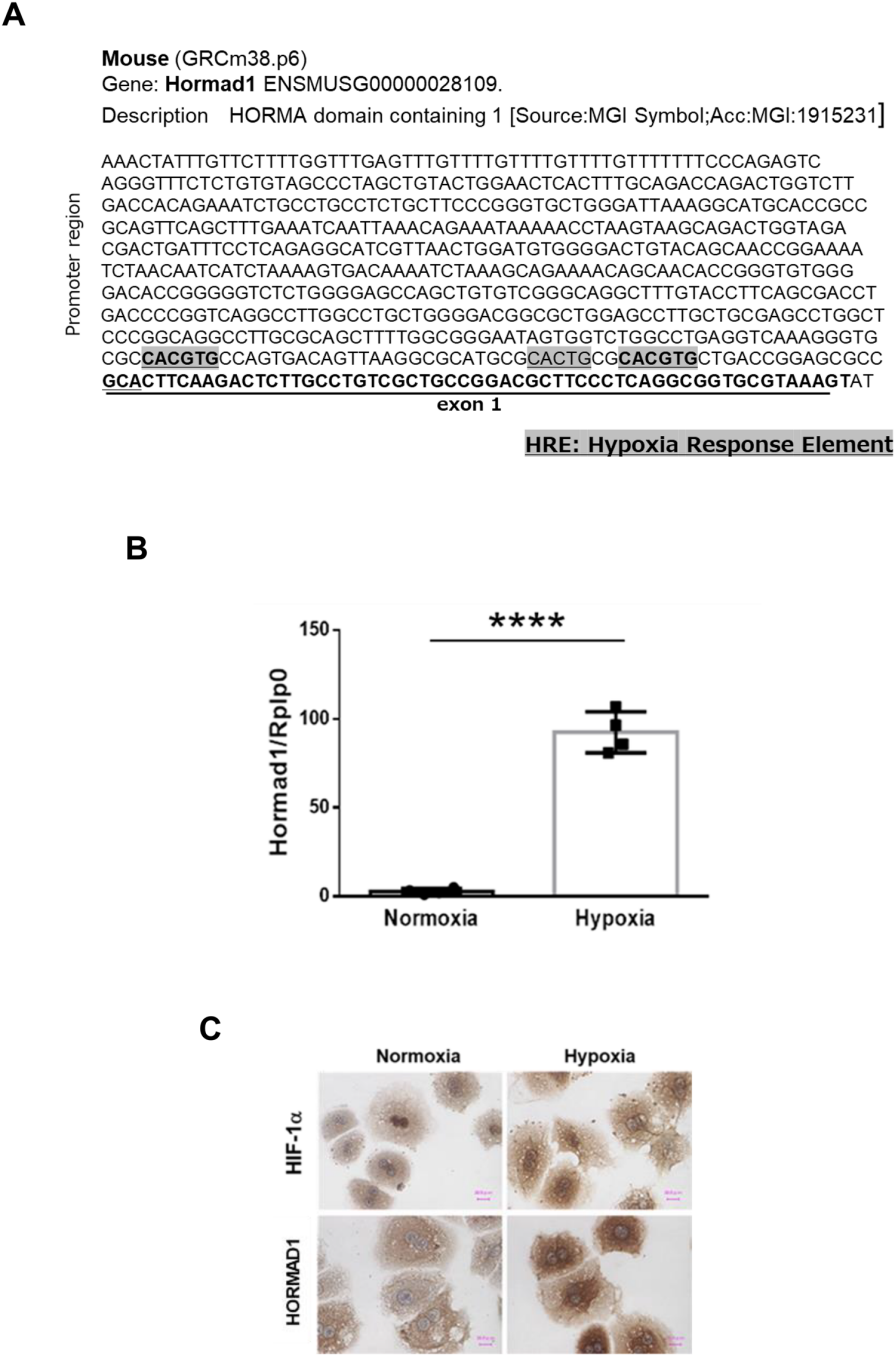

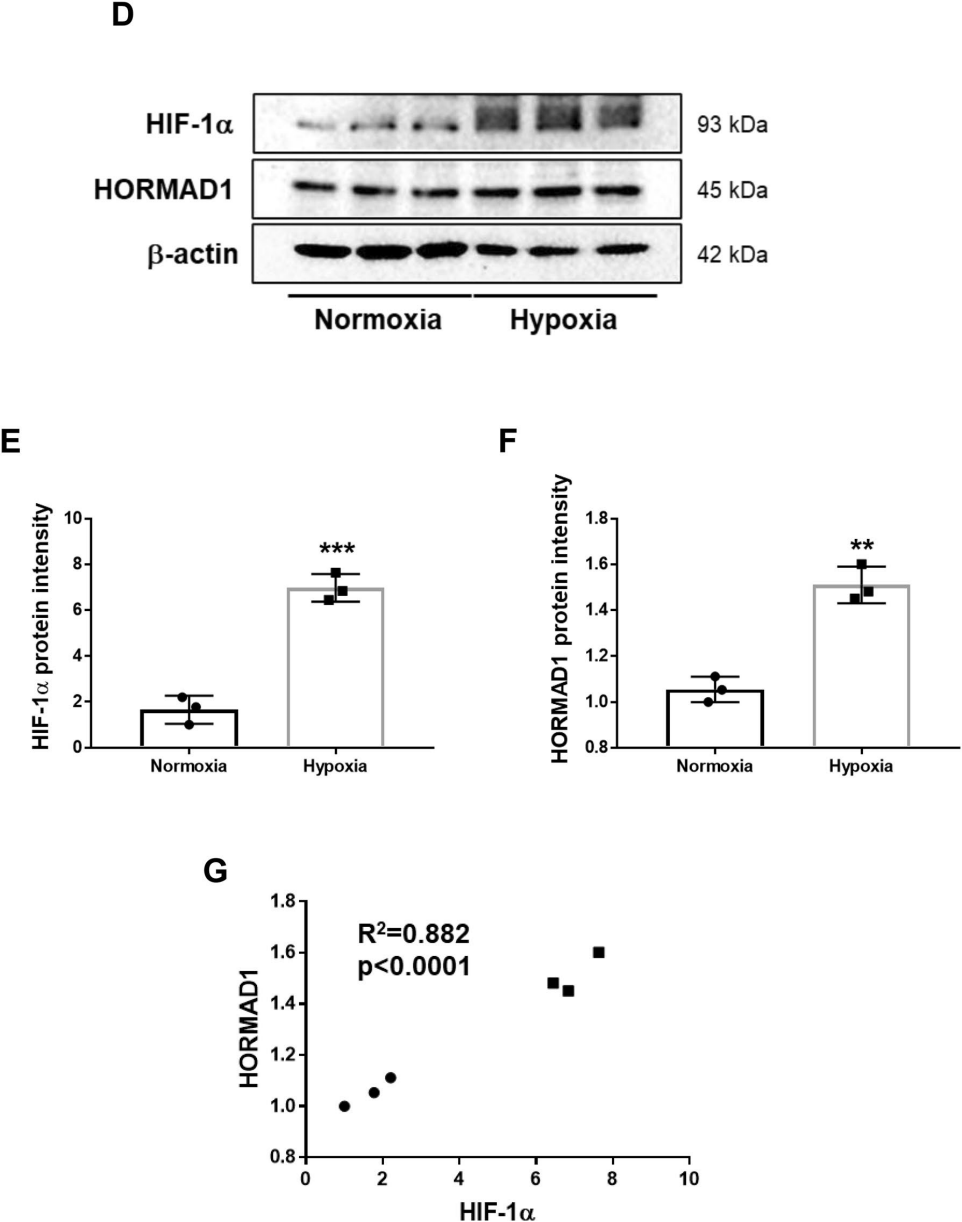

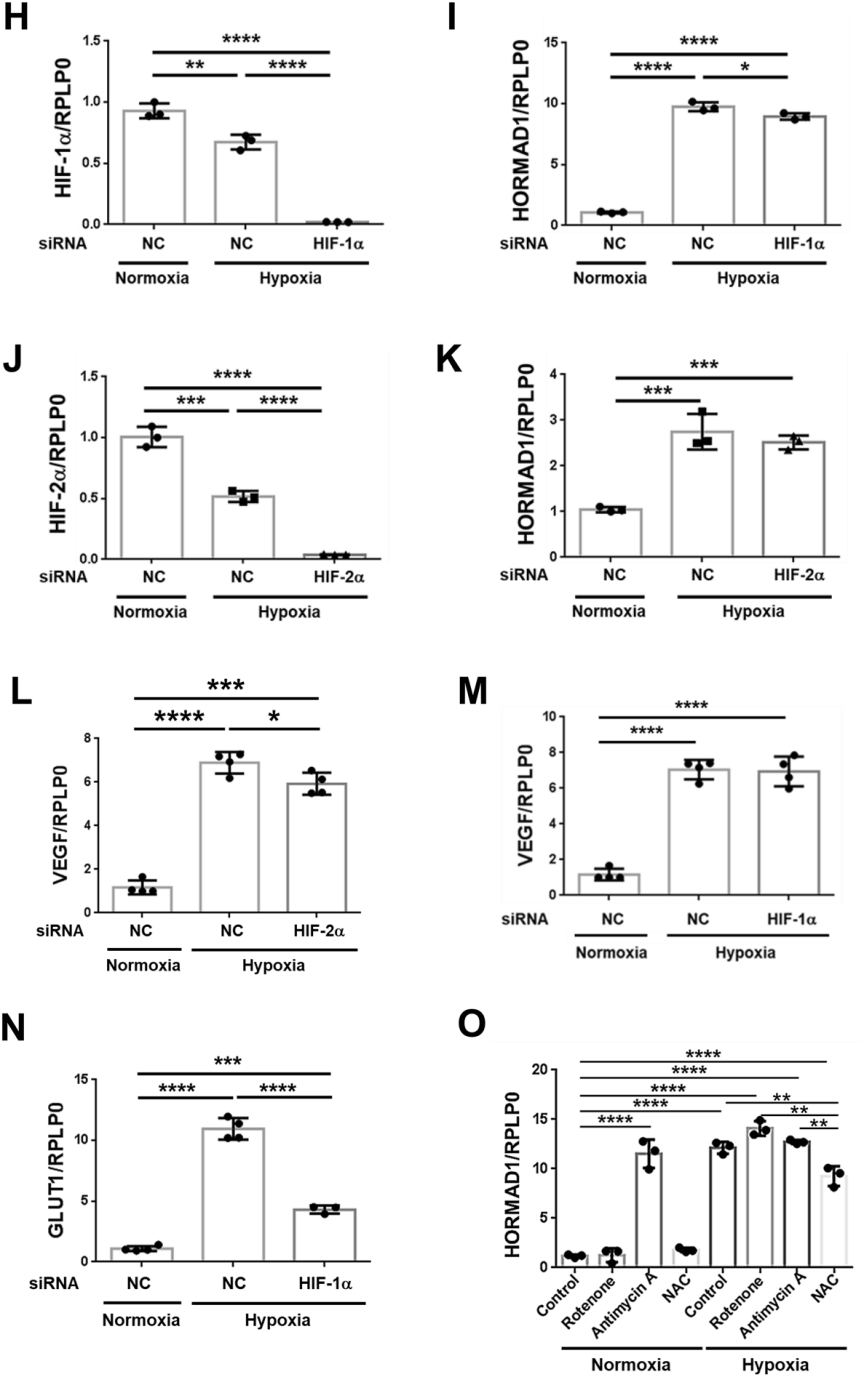
Hypoxia induces HORMAD1 expression in MPHs. (**A**) Three putative HREs containing the consensus sequence (A/G)CGTG within the mouse *Hormad1* gene. (**B**) *Hormad1* mRNA expression in MPHs was examined using qRT‒PCR. Isolated MPHs were exposed to hypoxia (1% O_2_) overnight. (**C**) Immunocytochemical staining for HIF-1α and HORMAD1 in MPHs cultured under normoxic or hypoxic conditions. Bars: 30 μm. (**D**) Western blot analysis of the total cell lysates of MPHs. (**E**) Effects of hypoxia on HIF-1α protein expression in MPHs. (**F**) Effects of hypoxia on HORMAD1 protein expression in MPHs. (**G**) Positive correlation between HIF-1α and HORMAD1 protein expression in MPHs (R^2^ = 0.882, *p* < 0.0001). (**H**) The efficacy of siRNAs targeting HIF-1α in reducing the *Hif-1α* mRNA expression level in MPHs. (**I**) The effect of the HIF-1α siRNA on the *Hormad1* mRNA expression level in MPHs. (**J**) The efficacy of the HIF-2α siRNA in reducing the *Hif-2α* mRNA expression level in MPHs. (**K**) The effect of the HIF-2α siRNA on the *Hormad1* mRNA expression level in MPHs. (**L**) The effect of the HIF-2α siRNA on the *Vegf* mRNA expression level in MPHs. (**M**) The effects of the HIF-1α siRNA on the *Vegf* mRNA expression level in MPHs. (**N**) The effect of the HIF-1α siRNA on *Glut1* mRNA expression levels in MPHs. (**O**) MPHs were treated with mitochondrial inhibitors of complex I (rotenone, 1 μM) and complex III Q_i_ sites (antimycin A, 1 μM) and the glutathione precursor *N*-acetylcysteine (NAC, 1 mM) to determine their effects on *Hormad1* mRNA expression. All data are presented as the means ± SD, and they are representative of at least three independent experiments. The significance of differences between groups was determined using unpaired Student’s *t test*s. Statistical comparisons were analyzed using one-way ANOVA with post hoc Bonferroni multiple comparison tests. *p < 0.05, **p < 0.01, ***p < 0.001, and ****p < 0.0001.

As HORMAD1 localized with the HIF-1α protein in fetal and livers of adult offspring from HFD-fed dams, we assessed whether *Hormad1* expression was directly activated by hypoxia. We identified three potential HIF-1 binding sites (hypoxia response elements; HREs) in the promoter region of the mouse *Hormad1* gene (Fig. 4A). Consistently, hypoxia markedly induced *Hormad1* mRNA expression in MPHs (Fig. 4B). Moreover, immunocytochemistry showed that hypoxia upregulated HORMAD1 protein expression in MPHs (Fig. 4C).

Hypoxia induced either HIF-1α (Fig. 4D,E) or HORMAD1 (Fig. 4D,F) protein expression in MPHs. The connection between HORMAD1 protein expression and HIF-1α protein expression suggested that both proteins are regulated by the same pathways (Fig. 4G).

However, the HIF-1 siRNA only slightly decreased hypoxia-induced *Hormad1* mRNA expression (*p* < 0.05, Fig. 4H,I), and the HIF-2α siRNA failed to alter *Hormad1* mRNA expression (Fig. 4J,K). The expression of glucose transporter isoform 1 (GLUT1)^38^ and VEGF^39^ are also known to be increased in HCC and are involved in tumorigenesis. Hypoxia induces the expression of these key proteins involved in the pathogenesis of HCC. Interestingly, the HIF-2α siRNA (Fig. 4L), but not the HIF-1α siRNA (Fig. 4M), slightly inhibited hypoxia-induced *Vegf* expression. However, the HIF-1α siRNA markedly inhibited *Glut1* mRNA expression (Fig. 4N). Then, we studied the effects of inhibitors of mitochondrial complex I (rotenone), the complex III Q_i_ site (antimycin A), and the glutathione precursor N-acetylcysteine (NAC) on *Hormad1* mRNA expression in MPHs (Fig. 4O). Rotenone (1 μM) did not alter *Hormad1* mRNA expression compared with the respective control under normoxic or hypoxic conditions, suggesting that mitochondria are not involved in the signaling regulating hypoxia-induced *Hormad1* mRNA expression (Fig. 4O). Antimycin A (1 μM), which acts at the principal site of ROS generation during hypoxia^40^, induced *Hormad1* mRNA expression compared with the normoxic control (Fig. 4O). The antioxidant NAC (1 mM) decreased hypoxia-induced *Hormad1* expression in cells treated with or without reagents. In summary, hypoxia-induced production of ROS, which are derived from nonmitochondrial sources, may be involved in the mechanism regulating *Hormad1* mRNA expression.

### Synchrotron radiation micro-CT assessment of the liver circulation

**Figure 5:**
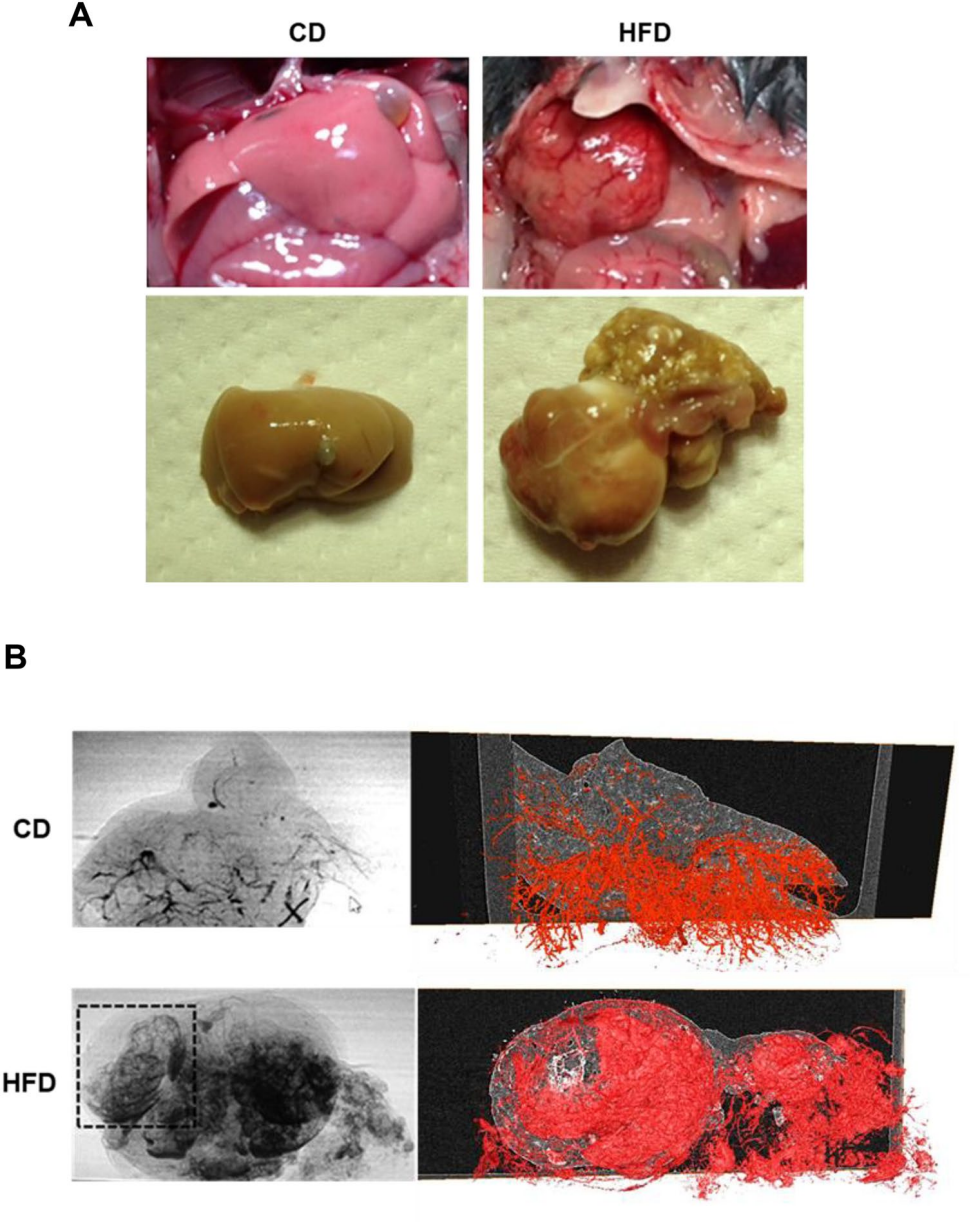

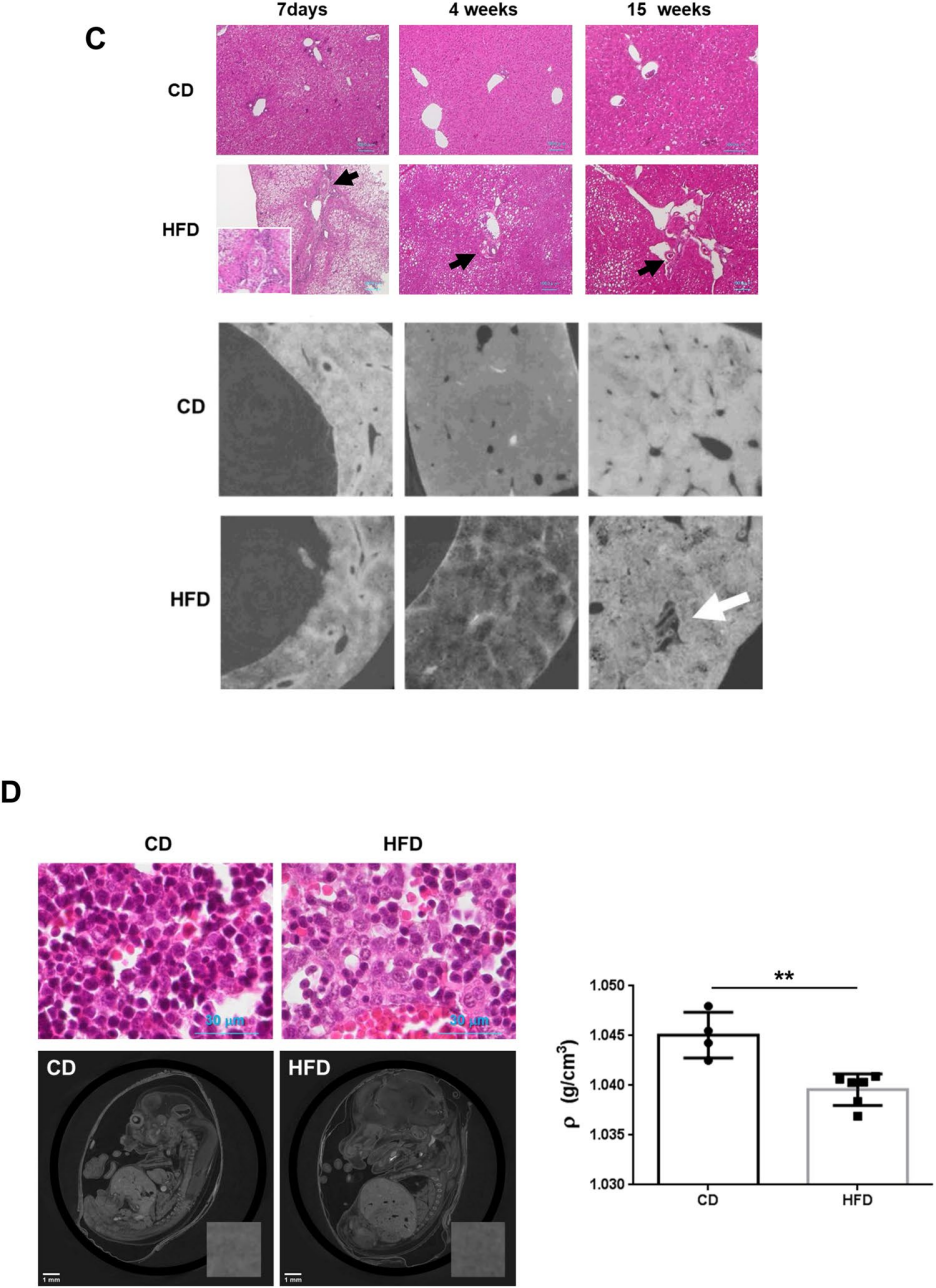
Synchrotron radiation micro-CT images show liver circulation in hepatic tumors and fetal livers. (**A**) Photographs of livers of 33-week-old offspring from CD- or HFD-fed dams (upper panels). Macroscopic images of livers after barium perfusion (lower panels). (**B**) CT fluoroscopy (left panels) and visualization of 3D vessels via synchrotron micro-CT using Amira software (version 1.4.0, https://www.thermofisher.com) (right panels) of livers from offspring. The square surrounded by the dotted line shows the typical basket pattern of tumor blood vessels. The computed tomography data from the offspring livers were uploaded to advanced 3D image processing and quantification software (Amira). (**C**) Hematoxylin and eosin staining (upper panels) and phase-contrast micro-CT imaging (lower panels) of livers of offspring at 7 days (CD; n = 7, HFD; n = 9), 4 weeks (CD; n = 6, HFD; n = 9) and 15 weeks (CD; n = 17, HFD; n = 11) of age. Black and white arrows show abnormal vascular structures, including large and thick hepatic arteries, in the livers from offspring of HFD-fed dams. Scale bars: 100 μm. (**D**) Hematoxylin and eosin staining of fetal livers (upper panels. Scale bars: 30 μm). Phase-contrast micro-CT imaging of fetuses (CD; n = 4, HFD; n = 6) at 14.5 dpc from CD- and HFD-fed dams. Scale bars: 1 mm. Hepatic tissue density (ρ) of offspring from CD- and HFD-fed dams. All data are presented as the means ± SD. Measured variables were log(e) transformed for statistical analyses. The significance of the differences between groups was determined using unpaired Student’s *t test*s. **p < 0.01.

Synchrotron micro-CT imaging performed at SPring-8 using contrast media showed architectural remodeling of the parenchyma in 33-week-old offspring from HFD-fed dams (Fig. 5A). The imaging results revealed the arterial basket pattern, which is one of the categories of vascular patterns of hepatocellular carcinoma (Fig. 5B)^41^.

On the other hand, phase-contrast X-ray imaging is an appropriate technique for observing the structure inside biological soft tissues, including the mouse fetus^42^, which are transparent to hard X-rays^43,44^. We assessed the structure of the livers of offspring at 7 days (CD; n = 7, HFD; n = 9), 4 weeks (CD; n = 6, HFD; n = 9) and 15 weeks (CD; n = 17, HFD; n = 11) of age using synchrotron-based phase-contrast micro-CT (Fig. 5C). The livers of offspring from HFD-fed dams had a lower density, consistent with fatty livers, even at 7 days after birth (Fig. 5C). In addition, phase-contrast micro-CT can detect an abnormal hepatic vascular structure, which consisted of arteriolar dilation and narrowing of the portal vein from 7 days to 15 weeks (Fig. 5C, white arrow), and these results were consistent with the large and thick hepatic arteries observed with hematoxylin and eosin staining (Fig. 5C, black arrows and inset in the upper left panel). As we detected histological changes in the livers of neonatal offspring from HFD-fed dams, we performed phase-contrast micro-CT of fetuses from CD- and HFD-fed dams (Fig. 5D). Hematoxylin and eosin staining showed fewer hematopoietic cells in the liver of fetuses from HFD-fed dams (Fig. 5D). Consistently, livers of fetuses from HFD-fed dams exhibited a significantly lower density (*p* < 0.01, Fig. 5D, Table 2), suggesting that maternal HFD consumption might induce fetal fatty liver, as reported in previous studies^45,46^.

**Table 2:** Liver volume, diameter of umbilical vein, diameter of ductus venosus and hepatic density in mouse fetuses measured by synchrotron radiation-based phase-contrast computed tomography at SPring-8.

Fetuses	V_Liver_ (mm^3^)	D_UV_ (μm)	D_UV_^3^ (mm^3^)	D_DV_ (μm)	LN (D_UV_^3^)/LN (V_Liver_)	ρ (kg/m^3^)
**CD**						
1	18.897	320.268	0.033	120.268	5.993	1.045
2	23.911	306.589	0.029	155.395	5.498	1.048
3	18.476	344.951	0.041	154.761	6.118	1.044
4	21.017	391.868	0.060	127.636	5.982	1.043
Mean	20.575	340.919	0.041	139.515	5.898	1.045
SD	2.486	37.492	0.014	18.223	0.274	0.002
**HFD**						
1	21.107	282.976	0.023	136.594	5.647	1.041
2	25.891	347.096	0.042	115.444	5.477	1.038
3	24.504	296.342	0.026	128.678	5.422	1.037
4	26.167	338.653	0.039	131.517	5.436	1.041
5	27.579	370.841	0.051	154.116	5.432	1.040
6	25.635	314.103	0.031	124.012	5.400	1.040
Mean	25.147	325.002	0.035	131.727	5.469	1.040
SD	2.213	33.112	0.011	13.107	0.091	0.002
p value	0.016	0.498	0.498	0.467	0.007	0.009

*V*_*Liver*_ liver volume, *D*_*UV*_ diameter of umbilical vein, *D*_*DV*_ diameter of ductus venous, *ρ* hepatic tissue density.

### Maternal HFD consumption induced fetal hepatomegaly without a compensatory increase in hepatic blood flow

**Figure 6:**
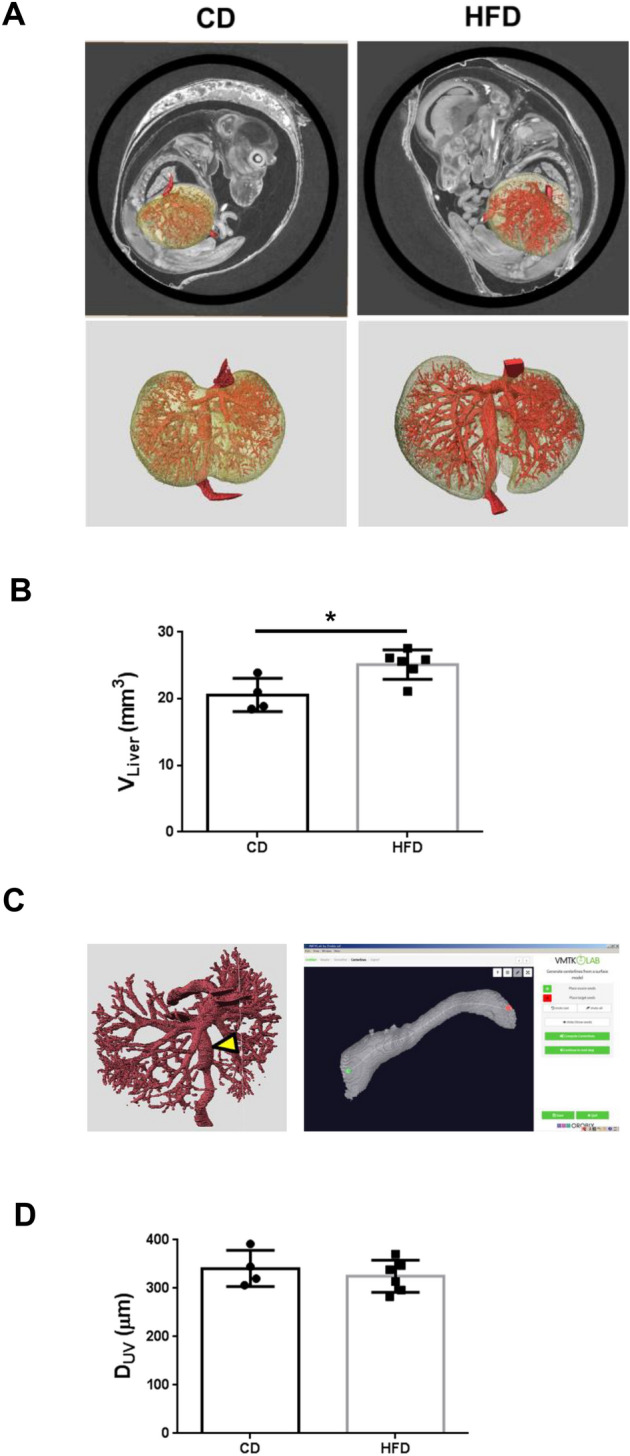

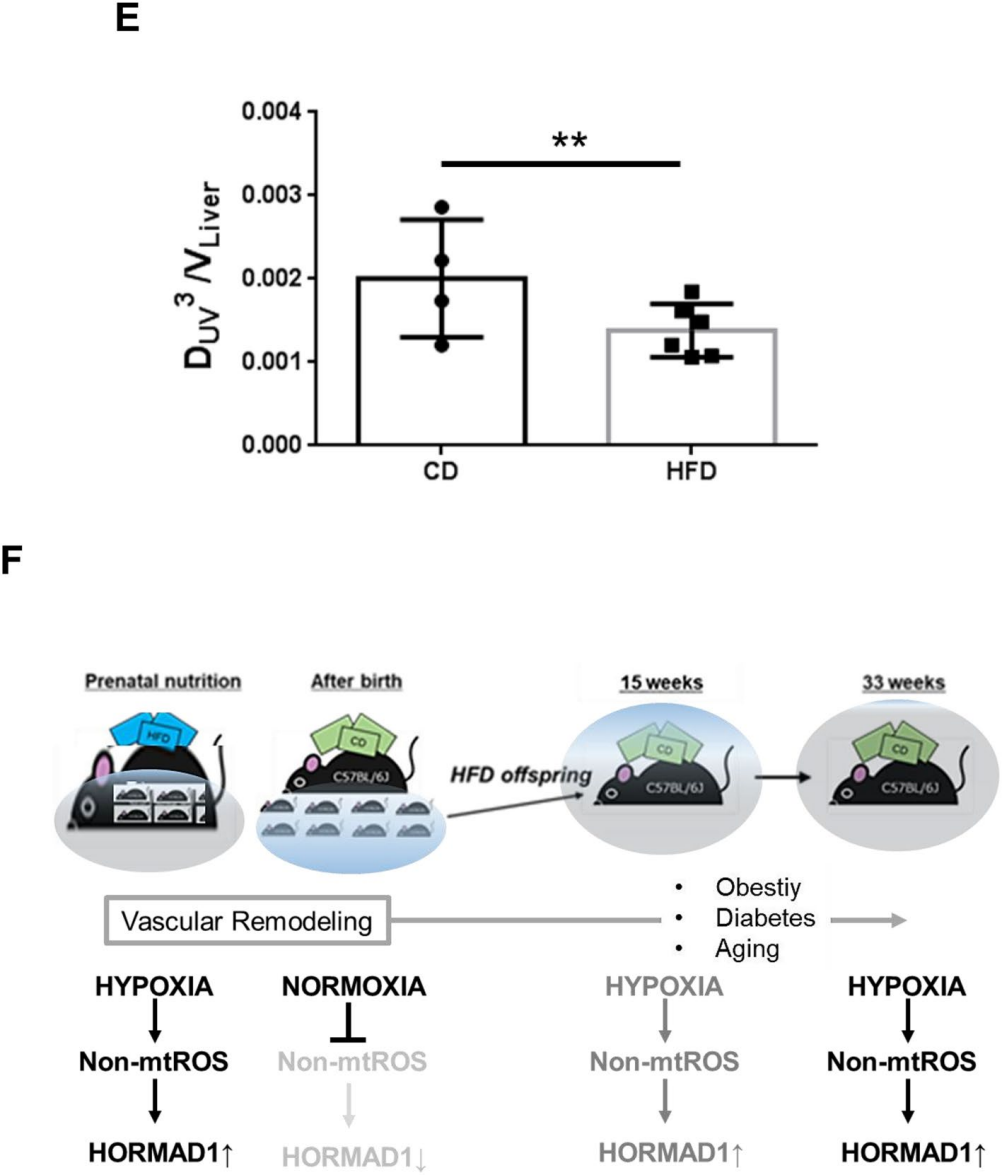
Maternal HFD consumption induced hepatomegaly without a compensatory increase in blood flow in fetuses. (**A**) Phase-contrast micro-CT images of fetuses (upper panels) were uploaded to advanced 3D image processing and quantification software (Amira, version 1.4.0, https://www.thermofisher.com) and 3D images of the fetal liver and intrahepatic vascular structure (lower panels) are presented. (**B**) Liver volume of fetuses (V_Liver_). The volume of the extracted liver was derived by forming multiple tetrahedrons with the center point as the apex in Amira. CD; n = 4, HFD; n = 6. (**C**) Hepatic vessels were extracted from fetuses using 3D image processing and quantification software (Amira). Intrahepatic blood vessels were extracted from the umbilical vein (yellow arrowhead) just before they entered the liver and branched. The blood flow rate was determined by deriving the vessel diameter, which is proportional to the multiplier of the vessel diameter according to Murray’s law (left panel). An open-source toolkit (Vascular Modelling Toolkit, version 1.4.0, VMTK, www.vmtk.org) was used to derive the vessel diameter semiautomatically (right panel). (**D**) Diameter of the umbilical vein (D_UV_) of fetuses. CD; n = 4, HFD; n = 6. (**E**) The blood flow rate is proportional to D_UV_^3^ per volume of fetal liver (V_Liver_). CD; n = 4, HFD; n = 6. All measured variables were log(e) transformed for statistical analysis. The significance of the differences between groups was determined using unpaired Student’s *t test*s. *p < 0.05 and **p < 0.01. (**F**) Maternal HFD consumption induces hepatomegaly without increasing hepatic blood flow in the fetus, resulting in hypoxia in the fetal liver. Hypoxia induces nonmitochondrial ROS production, leading to the upregulation of HORMAD1 in the fetal liver. After birth, breathing induced normoxia in the livers of offspring, attenuating the increased HORMAD1 expression. Obesity, diabetes and aging, which are known to induce hypoxia and ROS production, might increase HORMAD1 expression. The first hit in NAFLD/NASH/HCC development might be that fetal nutrition causes hepatic hypoxia. Thus, HORMAD1 expression is an indicator of tissue hypoxia resulting from ROS production.

We measured the liver volume of offspring using phase-contrast micro-CT (Fig. 6A,B, Table 2). Maternal HFD consumption significantly induced fetal hepatomegaly (*p* < 0.05, Fig. 6B, Table 2).

Immunohistochemistry revealed that maternal HFD consumption resulted in increased hepatic hypoxia and the expression of HIF-1α and HORMAD1 in offspring (Fig. 1D, Fig. 3A,C,G). In addition, hypoxia induced HORMAD1 expression in mouse hepatocytes via ROS production (Fig. 4). In the fetus, nutrients and oxygen from the placenta reach the fetus and fetal liver through the umbilical vein. After supplying the left hepatic lobe, the umbilical vein branches into the ductus venosus and the left portal vein. In the fetus, the left portal vein supplies the right hepatic lobe similarly to the portal vein, and the ductus venosus pumps blood from the umbilical vein directly into the systemic circulation via the inferior vena cava and right atrium. Considering the effects of fetal overnutrition on the fetal liver in this study, we thought that the most appropriate approach would be to compare its blood flow with that of the umbilical vein (Fig. 6C). We did not observe an increase in the diameter of the umbilical vein (Fig. 6D, Table 2). Blood flow is determined by the supplying vascular diameter due to Murrays’ minimum energy hypothesis^47^. Therefore, we studied the ratio of blood flow as the 3rd power of the diameter of the umbilical vein of the offspring per liver volume, indicating its lower ratio in offspring from HFD-fed dams, which suggests a lack of compensatory blood flow to account for the hepatomegaly (Fig. 6E).

## Discussion

This study is the first to report that gestational nutrients, not drugs or genetic modifications, cause NASH-based HCC in offspring. According to a recent study, maternal obesity increases the incidence of HCC in offspring via miR-27-3P^48^. However, maternal HFD consumption increased the incidence of diethylnitrosamine (DEN)-induced HCC in male offspring. In addition, female mice were fed a HFD from 1 to 3 months old before mating^48^. After male offspring from HFD-fed dams were injected with DEN, a genotoxic compound that is widely used to model liver tumorigenesis in rodents, at 15 days old, 50% survived for up to 10 months. In contrast, we fed dams a HFD after the observation of the plug until delivery to study the effect of maternal nutrients during pregnancy not including lactation. Coincidently, we realized that maternal HFD consumption caused NASH-based HCC in offspring without affecting the maternal liver. Additionally, the fatty liver observed in one-week-old offspring from HFD-fed dams prompted us to speculate that the trigger for NASH-based HCC might be present during the fetal period. A previous study showed that maternal Western-style diet-induced obesity (OB-WSD) for 2–9 years prior to conception and throughout pregnancy in adult female Japanese macaques induces hypoxemia and hepatic oxidative stress in fetuses^49^. Fetuses from OB-WSD mothers have lower O_2_ saturation, indicating some degree of fetal hypoxemia^49^. Switching OB-WSD mothers to the control diet prior to and for the duration of the next pregnancy restores the hypoxemia of fetuses in the next pregnancy, but incomplete normalization of the liver and serum metabolomes is observed in fetuses^49^. In the current study, the HFD was provided only during pregnancy, not prior to pregnancy or after delivery. However, the short duration of nutrient overload might induce the development of NAFLD/NASH/HCC in offspring from the neonatal period until adulthood. HFD feeding during pregnancy might be too short of a time period for the offspring to adapt to the metabolic changes in the intrauterine and neonatal periods. The previous studies described above^49^ that examined the effect of OB-WSD on fetuses have typically fed the animals a HFD before pregnancy and during lactation, which might cause adaptation of the fetuses to the maternal HFD-induced physiological changes, suppressing the development of NASH/HCC in the offspring. However, because only 20 days of HFD feeding after birth did not induce NASH/HCC in adult mice, fetuses should be more sensitive to nutrition during pregnancy, which is concurrent with the period of hepatic development. The metabolic changes related to fetal hepatic steatosis and hepatomegaly induced by OB-WSD are still present in offspring at 1 year of age^50^. In addition, prenatal fatty liver must provoke severe insulin resistance in the offspring of HFD-fed dams beginning early in life, accompanying the inhibition of insulin-stimulated glucose uptake. CD-feeding with insulin resistance after birth might lead to malnutrition, such as kwashiorkor and marasmus^51^, and exacerbate steatohepatitis.

The effect of the HFD load in utero rather than in adulthood also indicates potential contributing factors other than the nutritional load. A recent study reported that decreased portal circulation by congenital portosystemic shunt (PSS) augments fibrosis and the ductular reaction in NASH in C57BL/6J mice^52^. PSS induces hypoxia due to disrupted intralobular perfusion and is an important factor aggravating liver fibrosis in individuals with NASH^52^. Similarly, in our model, maternal HFD consumption induced fetal hepatomegaly without a compensatory increase in the blood supply, resulting in hypoxia in the fetal liver. In fact, we have frequently observed PSS in the livers of offspring of HFD-fed dams. According to another study, HFD feeding for 16 weeks from eight weeks of age induces liver hypoxia and leads to HIF-1α upregulation in NASH mice^53^. Therefore, we speculate that maternal HFD-induced hypoxia in the fetal liver might play an important role in the development of NASH/HCC.

We found that the fetuses from HFD-fed dams had hepatomegaly without a compensatory increase in the blood supply. Under normal conditions, 75–80% of hepatic blood flow is of umbilical origin^54^. Therefore, umbilical venous blood flow represents the quantity of oxygen and nutrients reaching the fetus. Because the oxygen consumption of the liver in fetal sheep accounts for 20% of the total fetal oxygen consumption, during hypoxia, total liver blood flow and the umbilical venous contribution both decrease by 20% to reduce its oxygen consumption^54^. Pregnancies with pregestational diabetes show a mismatch between greater fetal growth (macrosomia) and uncompensated liver perfusion (nutrient supply) after 30 weeks of pregnancy^55^. Moreover, the maternal prepregnancy body mass index alters the fetal blood flow distribution to modulate fetal handling of glucose^56^. Maternal HFD-induced fetal hepatomegaly might respond to hypoxia by maintaining the diameter of the umbilical vein, as observed in fetuses from CD-fed dams, resulting in lower oxygen consumption in the liver. The umbilical vein connects to the left portal vein, which connects to the ductus venosus and carries the blood to the inferior vena cava. We also did not observe a difference in the diameters of the ductus venosus between HFD-fed dams and CD-fed dams (Table 2). As hepatic oxygen consumption accounts for 20% of the total fetal oxygen consumption, hepatic venous blood is well oxygenated and provides an important source of oxygen for fetal tissues^54^. Therefore, fetal hepatic hypoxia will result in the failure to carry sufficient oxygen to other fetal tissues.

Synchrotron radiation micro-CT showed that maternal HFD consumption induced fetal fatty liver (Table 2). Hypoxia is tightly associated with lipid homeostasis and increases cellular lipid deposition^57^. Hepatocyte-specific HIF-1α expression is a determinant of lipid accumulation and liver injury in alcohol-induced steatosis in mice^58^. A HFD containing lipids may promote the β-oxidation of free fatty acids, which requires a considerable amount of oxygen, leading to the stabilization of HIF-1α subunits by the inhibition of PHDs. Thus, a loop involving HIF-1α and lipid deposition in the liver might establish a vicious cycle that augments hypoxia in individuals with NAFLD.

Because the ductus venosus also supplies oxygen and nutrients to the heart and brain, the oxygen supply for fetal tissues will depend on ductus venous blood flow when maternal HFD consumption induces fetal hepatic hypoxia. Thus, oxygen redistribution in the fetus might lead to vascular remodeling, such as a ductus venosus shunt^59^, accompanied by narrowing of the portal vein and dilation of the hepatic artery of the offspring.

We assessed gene expression in fetal livers. Among the 430 DEGs identified, including 146 upregulated and 284 downregulated genes in the fetal livers from HFD-fed dams compared with those from CD-fed dams (Table 1), we found that HORMAD1 was significantly upregulated in the livers of fetuses from HFD-fed dams (Table 1). HORMAD1 was originally cloned as a CT antigen^25^. HORMAD1 was recently shown to compromise DNA mismatch repair in cancer cells by interacting with the MCM8-MCM9 complex and reducing chromatin binding of MLH1, the key component of the DNA mismatch repair machinery^32^. Aberrant expression of HORMAD1 is associated with genomic instability in triple-negative breast cancers^28,29^, lung cancers^30,31^, and ovarian cancers^32,33^. Promoter hypomethylation is known to upregulate HORMAD1 in various cancers^30,60^. We identified three putative HREs in the mouse *Hormad1* promoter region. Moreover, hypoxia induced HORMAD1 mRNA and protein expression in MPHs. However, the HIF-1α siRNA slightly suppressed hypoxia-induced *Hormad1* mRNA expression. In addition, the HIF-2α siRNA failed to inhibit hypoxia-induced *Hormad1* mRNA expression. Hypoxia induces VEGF expression via ROS production independent of HIF-1 in HEK293T or C6 cells^61^. Therefore, we studied the effects of ROS on regulating *Hormad1* mRNA expression by inhibiting mitochondrial respiration (rotenone), inducing mitochondrial ROS production (antimycin A^40,62,63^) and using an antioxidant (NAC). Antimycin A induced *Hormad1* mRNA expression under normoxic conditions. However, rotenone, which inhibits mitochondrial ROS production, did not inhibit hypoxia-induced *Hormad1* mRNA expression. Finally, NAC significantly inhibited hypoxia-induced *Hormad1* mRNA expression. In summary, hypoxia induces *Hormad1* mRNA expression via nonmitochondrial ROS production. Maternal OB-WSD before and during pregnancy increases hepatic triglyceride accumulation in fetuses without impairing mitochondrial function in fetal livers^49^. In the current model of maternal HFD-induced NASH/HCC, mitochondrial dysfunction might not be involved in the induction of HORMAD1 expression in the livers of offspring. Most likely, hemodynamic regulation in response to maternal HFD-induced hypoxia might induce ROS production rather than metabolic dysfunction in hepatic mitochondria^64^. ROS generation via NOX contributes to the proliferation of hepatic oval cells^65^, which are progenitor cells associated with an increased risk of developing HCC^66^. We observed oval cell proliferation in peritumoral regions of 33-week-old offspring from HFD-fed dams (Supplementary Fig. 4). NOX might be a potential site for hypoxia-induced ROS production in the livers of offspring from HFD-fed dams (Fig. 6F).

We detected abnormalities in the vascular system prior to fibrosis in the neonatal livers of offspring from HFD-fed dams. Hypoxia produces profibrotic mediators in hypoxic hepatocytes^67^. Fetal hepatic hypoxia might induce the production of profibrotic factors around the portal vein, resulting in the development of fibrosis around the portal vein after birth. Recently, congenital portosystemic shunts were shown to decrease portal circulation, which induces hypoxia and augments fibrosis in mice^52^. However, we also obtained evidence of NASH/HCC in offspring from HFD-fed dams of C57BL/6N mice, which have no PSS^59^. Notably, abnormal vascular structures, such as large hepatic arteries and narrowing of the portal veins, were observed in the livers of C57BL/6N offspring from HFD-fed dams. Therefore, portosystemic shunting is not essential for the development of maternal HFD-induced NASH/HCC in offspring and is a secondary phenomenon caused by maternal overnutrition. Mitochondrial redox abnormalities might facilitate maternal HFD-induced ROS production in C57BL/6J mice, leading to the abnormal vascular structure and hypoxic liver injury.

Both HORMAD1 and VEGF expression are increased by hypoxia via ROS production. Hyperoxia also causes a significant increase in iNOS levels paralleled by a significant decrease in intracellular glutathione (GSH) levels in rat liver^68^. In addition, NAC (1 mM) blocked the hyperoxia-induced increase in iNOS levels and restored GSH levels^68^. Therefore, either hypoxia or hyperoxia might cause ROS-mediated liver injury, and NAC might reverse oxygen redistribution-induced liver injury. In the current model, the HFD load during gestation induced hypoxia in the fetal liver because of the loss of a compensatory increase in blood flow to accommodate fetal hepatomegaly. Fetal hepatic hypoxia might induce VEGF expression via ROS production, leading to remodeling of the vascular structure in the fetal liver. Conversely, neonatal hepatic hyperoxia mediated by larger and thicker hepatic arteries in zone 1 in the livers of the offspring from HFD-fed dams might induce ROS production, which also causes an increase in the expression of hypoxia-related genes. Thus, either hypoxia in zone 3 or hyperoxia in zone 1 potentially induces ROS in the livers of offspring hemodynamically. Oxygen redistribution, such as hypoxia and hyperoxia, is considered a pathological trigger of NASH/HCC^69^. Drastic changes in the oxygen status during the fetal period and after birth may be the pathophysiological basis of maternal HFD-induced NASH/HCC in offspring. HORMAD1 expression indicates the presence of hypoxia or tissue ROS production in the liver, as well as in some cancers^70,71^. HORMAD1 might be a biomarker for NASH/HCC and a therapeutic target, especially for fetal programming of nutrition-related chronic diseases.

## Methods

### Experimental design

All animal experiments conformed to the National Institutes of Health Guide for the Care and Use of Laboratory Animals and were approved by the Research Center for Animal Life Science at Asahikawa Medical University. The animal experiments conformed to the protocols, including ethical treatment, reviewed and approved by the Institutional Animal Care and Use Committee of Asahikawa Medical University (IACUC Approval No. R3-108). The sample sizes for the animal studies were guided by a previous publication that studied the effects of maternal HFD consumption on NASH in offspring^72^. All mice were housed with free access to chow and water on a 12-h light/dark cycle. For timed mating, females that were breeding were checked for vaginal plugs every morning, and if present, the time point was set to day post-coitum (dpc) 0.5. Then, the female C57BL/6J mice were randomly divided into two groups fed either a control diet (CD; 4.3% kcal fat, 19.2% kcal protein, and 67.3% kcal carbohydrate, D12450J, Research Diets Inc., New Brunswick, NJ) or a high-fat diet (HFD; 60% kcal fat, 20% kcal protein, and 20% kcal carbohydrate; D12492, Research Diets Inc.) during gestation. During lactation, all dams were fed the control diet. Because of the lack of significantly different phenotypes of liver pathology between male and female offspring and the usage of MPHs from male mice, most of the data are presented from experiments using male offspring. We used littermates born to some dams from each CD and HFD group that gave birth to 6 or more offspring to exclude the effect of the number of offspring on growth. After weaning, the male offspring were fed the control diet, generating two groups: CD and HFD. At 15 weeks of age, male offspring were assessed for body weight, and FBS, HbA1c, NEFA, and ALT levels, as well as systolic blood pressure (SBP). We performed two series of studies. First, we performed histological and biochemical studies, including the detection of hypoxia, immunohistochemical staining, and western blots for HIF-1α and HODMAD1 levels in mouse tissue sections (CD; n = 16 offspring from 4 dams, HFD; n = 15 offspring from 4 dams). Second, we measured the liver volume, diameter of the umbilical vein and tissue density of fetal livers (CD; n = 4 offspring from one dam, HFD; n = 6 offspring from two dams) and the tissue density of the livers of offspring at 7 days (CD; n = 7 offspring from 4 dams, HFD; n = 9 offspring from 4 dams), 4 weeks (CD; n = 6 offspring from 3 dams, HFD; n = 9 offspring from 4 dams) and 15 weeks (CD; n = 17 offspring from 7 dams, HFD; n = 11 offspring from 5 dams) of age using synchrotron-based phase-contrast micro-CT imaging at SPring-8. We performed the histological examination and micro-CT imaging using synchrotron radiation of 33-week-old offspring of HFD-fed dams to explore the malignant potential of this mouse model.

### Histological analysis

Three-micrometer-thick paraffin-embedded liver tissues and fetuses were stained with hematoxylin and eosin and Masson’s trichrome (blue = collagens; red = erythrocytes and cytoplasm; dark purple/black = nuclei). The stained sections or fetuses were observed and visualized using a light microscope system (BZ-8100; Keyence, Osaka, Japan. https://www.keyence.com).

### Immunohistochemistry

Immunohistochemical staining was performed as previously described^52,73^. Briefly, formalin-fixed sections were deparaffinized and then microwaved at 100 °C for 30 min to retrieve antigens. Sections were incubated with 3% hydrogen peroxidase for 10 min to block endogenous peroxidase activity. Then, the specimens were incubated overnight with the primary antibodies, rinsed with PBS, and subsequently incubated with ENVISION + (Dako, Carpinteria, CA) for 30 min. The reactions were visualized with 3,3′-diaminobenzidine tetrahydrochloride after 1–3 min, revealing brown staining. The cell nuclei were then counterstained with hematoxylin. Immunohistochemistry was performed with a rabbit polyclonal anti-HIF-1α antibody (1:400) (NB100-479, Novus Biologicals, Centennial, CO, USA), a rabbit polyclonal anti-HORMAD1 antibody (1:400) (28719-1-AP, Proteintech Group, Inc., Rosemont, IL, USA), and an anti-cytokeratin 19 (CK19) antibody (provided by Dr. Atsushi Miyajima, Institute for Quantitative Biosciences, The University of Tokyo, Tokyo, Japan). The stained sections were observed and visualized using a light microscope system (BZ-8100; Keyence). Quantification of immunohistochemical images was performed with Fiji Image J (US National Institutes of Health, Bethesda, MD, USA, imagej.net/Fiji, v.1.52) as described in the previous study^52^.

### Detection of liver hypoxia

After pimonidazole, a 2-nitro-imidazole, is delivered in vivo*,* it binds to thiol groups of proteins in tissue with oxygen tensions less than 10 mmHg, which can be detected using commercially available anti-pimonidazole antibodies^74^. For the detection of hypoxic conditions in mice, pimonidazole (60 mg/kg, CAS# 70132-50-2, Cayman Chemical Company, Ann Arbor, MI, USA) was injected intraperitoneally 1 h before the animals were euthanized^74^. Staining was performed using Hydroxyprobe (Pharmacia International, Belmont, MA, USA) according to the manufacturer’s instructions^75^. The stained sections were observed and visualized using a light microscope system (BZ-8100; Keyence).

### Microarray

The microarray was performed as described in a previously published study^76^. Total RNA was extracted with an RNeasy Mini Kit (Qiagen, Hilden, Germany) according to the manufacturer’s instructions. Cyanine-3 (Cy3)-labeled cRNA was prepared from 0.2 µg of RNA using the Low Input Quick Amp Labeling Kit (Agilent) according to the manufacturer’s instructions, followed by RNAeasy column purification (Qiagen). Dye incorporation and the cRNA yield were assessed with a NanoDrop ND-1000 spectrophotometer. Cy3-labeled cRNA (0.6 µg, specific activity > 25.0 pmol Cy3/µg cRNA) was fragmented at 60 °C for 30 min in a reaction volume of 25 µl containing 1 × fragmentation buffer (Agilent) and 2 × blocking agent (Agilent) according to the manufacturer’s instructions. Upon completion of the fragmentation reaction, 25 µl of 2 × GE Hybridization Buffer HI-RPM (Agilent) were added to the fragmentation mixture and hybridized to a SurePrint G3 Mouse GE microarray 8 × 60 K Ver. 2.0 (G4858A #74809, Agilent Technologies) for 17 h at 65 °C in a rotating Agilent hybridization oven. After hybridization, microarrays were washed for 1 min at room temperature with GE Wash Buffer 1 (Agilent) and for 1 min with 37 °C GE Wash buffer 2 (Agilent) and then dried immediately. Microarray slides were scanned immediately after washing using the Agilent DNA Microarray Scanner (G2539A) with the one color scan setting for 1 × 60 K array slides (scan area 61 × 21.6 mm, scan resolution 3 µm, dye channel set to green, and green PMT set to 100%). The scanned images were analyzed with Feature Extraction Software 11.0.1.1 (Agilent) using the default parameters (protocol AgilentG3_GX_1Color and grid: 074809_D_F_20150624) to obtain background-subtracted and spatially detrended processed signal intensities. Data were normalized and filtered with three filters using GeneSpring software 12.1 (Agilent Technologies. https://www.agilent.com). Briefly, the raw data were normalized to their 75th percentile values. In the first filter, when the value of a probe was lower than the 20th percentile value in any sample, the probe was excluded. In the second filter, when the flag of a probe was compromised in any sample, the probe was excluded. In the last filter, when the value (log scale) of CV was greater than 50% under both conditions, the probe was excluded. After filtering, control probes were excluded, and log scale values were transformed into normal scales. The microarray data have been deposited in the Gene Expression Omnibus (GEO) database under number GSE183360.

Data are presented using GeneSpring software 12.1 (Agilent Technologies. https://www.agilent.com).

### Western blotting

Total cellular extracts from mouse liver tissues and mouse primary hepatocytes were prepared as described previously^75^. Western blotting was conducted using 4–12% Novex NuPAGE Tris–glycine gels (Invitrogen) for HIF-1α and HORMAD1 (Proteintech Group, Inc.) under reducing conditions. After the proteins were transferred onto Hybond-P PVDF membranes (Amersham Biosciences Co., Piscataway, NJ, USA), the membranes were incubated with a rabbit polyclonal anti-HIF-1α antibody at a dilution of 1:500 (NB100-479, Novus Biologicals USA) or a rabbit polyclonal anti-HORMAD1 antibody (28719-1-AP, Proteintech Group, Inc., Rosemont, IL, USA) at a dilution of 1:1000 at 4 °C overnight, followed by an incubation with a peroxidase-conjugated secondary antibody (dilution 1:50,000) (Amersham), and visualized with an enhanced chemiluminescence (ECL) system (Amersham ECL Prime). For loading controls, the same membranes were stripped and reprobed with a β-actin antibody at a dilution of 1:5000 (A5316, Sigma–Aldrich, St. Louis, MO, USA). As the levels of β-actin were affected by hypoxia exposure, PVDF membranes were stained with a Coomassie brilliant blue solution (CBB, 0.1% R250, 25% isopropanol, 10% acetic acid) solution for 5 min and washed with double-distilled water to confirm equal protein loading^77^. The protein intensities of the HIF-1α and HORMAD1 bands were processed using ImageJ software for densitometry analysis.

### Isolation and culture of MPHs

MPHs were isolated from 8-week-old male C57BL/6J mice using previously described methods^78^. The viable hepatocyte population was further purified by Percoll gradient centrifugation. Freshly isolated primary hepatocytes were plated in collagen-coated 6-well plates (IWAKI, Tokyo, Japan) at a density of 5 × 10^5^ cells per well and cultured with high-glucose Dulbecco’s modified Eagle’s medium (DMEM) (Gibco, 11965092, Carlsbad, CA, USA) supplemented with 10% fetal bovine serum, insulin-transferrin-selenium supplement (Gibco), 10^–7^ M dexamethasone (Sigma–Aldrich, Inc., St. Louis, MO) and antibiotics. Cells were allowed to adhere overnight before switching to fresh medium and were exposed to reagents under normoxic (21% O_2_) or hypoxic (1% O_2_) conditions for 24 h before being harvested for experiments, as previously described^75^.

### Quantitative RT-PCR

Total RNA was extracted from mouse livers and primary hepatocytes from each group using an RNeasy mini kit (Qiagen, CA) according to the manufacturer’s instructions. cDNA synthesis was performed with a High-Capacity cDNA Reverse Transcription Kit with RNase Inhibitor (Applied Biosystems, Carlsbad, CA). Each cDNA sample was analyzed for gene expression using quantitative real-time PCR with a fluorescent TaqMan 57-nuclease assay and a sequence detection system (Prism 7300, Applied Biosystems, CA). TaqMan real-time PCR was performed using 2 × TaqMan Master Mix and 20 × assay-on-demand TaqMan primers and probes (Applied Biosystems, Thermo Fisher Scientific, Foster City, CA). The analysis was performed with ABI Prism 7300 SDS software (version 1.4.1, Applied Biosystems, https://www.thermofisher.com/jp/ja/home/technical-resources/software-downloads/applied-biosystems-7300-real-time-pcr-system.html). Unlabeled specific primers were purchased from Applied Biosystems to detect the mouse *Hif1α* gene (assay ID: Mm00468869), mouse *Hif2α* (assay ID: Mm01236112_m1) mouse *Hormad1* gene (assay ID: Mm00471448) and mouse ribosomal protein lateral stalk subunit P0 (*Rplp0*) gene (assay ID: Mm 00,725,448). After an initial incubation for 2 min at 50 °C and 10 min at 95 °C, the samples were cycled 55 times at 95 °C for 15 s and 60 °C for 1 min. For the quantitative analysis, the cDNA content of each sample was normalized to the levels of the housekeeping gene *Rplp0* using the comparative CT method.

### Immunocytochemistry

Primary hepatocytes were cultured on collagen type 1-coated four-chamber glass slides (BD Biocoat Collagen I, BD Biosciences, Bedford, MA, USA). After an overnight incubation under normoxic or hypoxic conditions, the cells were fixed with 100% ethanol for 10 min and incubated with a rabbit polyclonal anti-HIF-1α antibody (1:400) and a rabbit polyclonal anti-HORMAD1 antibody (1:400) at 4 °C overnight. Then, the cells were rinsed with PBS and subsequently incubated with a horseradish peroxidase-labeled polymer-conjugated secondary antibody (Envision, Dako, Carpinteria, CA) for 30 min at room temperature. The reactions were visualized with 3,3′-diaminobenzidine tetrahydrochloride after 2–3 min, revealing brown staining. The stained sections were observed and visualized using a light microscope system (BZ-8100; Keyence).

### siRNA transfection

Lipofectamine RNAiMAX transfection reagent (Thermo Scientific) was mixed with siRNAs (Dharmacon, ON-TARGETplus siRNA, Horizon Discovery, a PerkinElmer company, Cambridge, GB, Supplementary Table 2) in Opti-MEM reduced serum medium (Gibco) according to the manufacturer’s instructions, and cells were used in experiments 24 h later.

### Liver sample preparation for the synchrotron radiation micro-CT analysis

The contrast medium was prepared using a previously published protocol^79^. Briefly, the contrast medium consisted of 40% wt/vol (in distilled water) BaSO_4_ and 8% wt/vol gelatin. The abdomen and thorax were cut by creating a midline incision extending from the pubic symphysis to the jugulum under anesthesia. Three hundred units of heparin were injected into the beating left ventricle of the heart using a 24-gauge catheter. The right atrium was then cut, and the mouse was perfused first with 42 °C prewarmed heparinized saline (100 IU/ml heparin in 0.9% NaCl) and then with contrast medium (42 °C). Immediately after perfusion, cold saline (< 4 °C) was gently poured into the abdominal cavity. The mouse was carefully placed in an ice bucket for 10 min to solidify the contrast medium. Thereafter, the liver, which was filled with contrast medium, was removed and stored in a freezer. The sample exclusion criteria were determined before the CT scan for technical failures, such as misinjection of the contrast medium in the left ventricle or insufficient perfusion.

### Imaging of liver samples

Samples were visualized using the synchrotron micro-CT system in BL20B2 at SPring-8^79^ (Supplementary Fig. 5A). The synchrotron radiation beam is produced by deflecting the electron beam with a bending magnet, and the flux is much higher than that of laboratory X-ray sources. The X-ray was monochromatized with a Si (111) double crystal monochromator, and the beam then irradiated the samples. As the X-ray beam was approximately parallel, each horizontal line corresponded to a slice position along the rotation axis, and images of multiple slices (the slice pitch was equal to the pixel size, namely, the “cubic voxel”) were easily obtained in one rotation (3D-CT).

A high-resolution image detector (Beam monitor 5 and ORCA-Flash4.0; Hamamatsu Photonics, Hamamatsu, Japan) was used for radiographic imaging^80^. The format of the 3D image was 2048 × 2048 × 1320 pixels, with cubic voxels of 15.5^3^ μm^3^ (3.72 × 10^−6^ mm^3^). The scintillator, which converts the X-ray beam to visible light, was a 25-μm layer of GADOX (Gd_2_O_2_S: Tb^±^, P43). The X-ray energy was 37.6 keV, and the exposure time per projection was 100 ms.

For each sample, 1800 radiographic images were acquired over an angular range of 0°–180° within 3 min. In this study, the liver sample was placed on the rotating stage in an insulated chamber with double Kapton and Styrofoam walls and then scanned while the cryogenic temperatures were maintained using dry ice. After scanning, we reconstructed the CT images from the radiographic images using a conventional tomography algorithm and convolution back projection.

### X-ray phase tomography using a grating interferometer

X-ray phase tomographic measurements were performed at bending magnet beamline BL20B2 in SPring-8, Hyogo, Japan. An X-ray grating interferometer composed of a phase grating G1 and an absorption grating G2 was used to retrieve phase information (Supplementary Fig. 5B). The principle of the grating interferometer is described elsewhere^81^. The X-ray grating interferometer was set between a sample and an X-ray imaging detector, as shown in Supplementary Fig. 5B. G1 was composed of nickel with a pattern thickness of 3.53 μm, which was designed to generate a π/2 phase shift at an X-ray energy of 20 keV. G2 was composed of gold and its pattern thickness was greater than 20 μm. The grating pitch in both gratings was 2.4 μm. The distance between G1 and G2 was defined by the fractional Talbot distance. The 5th-order fractional Talbot distance (*p* = 5/2) was chosen as an interferometric condition. The fractional Talbot distance depended on the Talbot order *p*, the grating pitch and the X-ray energy. In this experimental setup, the fractional Talbot distance was calculated to be 232 mm. In preparation for the tomographic measurement, a sample was placed in a designated cylindrical container made of polypropylene with inner and outer diameters of 12 mm and 13 mm, respectively. When placing the sample in the container, an agarose gel with a concentration of 2% was used to fix the sample in the container. The sample in the container was measured within a specially designed water cell filled with pure water to avoid strong artifacts due to the drastic phase shift at the outer wall of the container. A visible light conversion-type X-ray imaging detector was used to detect projection images. It was composed of an AA60 beam monitor with a P43 scintillator (Hamamatsu Photonics) and a C13949-50U high-definition CMOS camera (Hamamatsu Photonics). In this case, the effective pixel size and the effective field of view were 3.47 μm and 14.2 mm in width, respectively. In the X-ray phase tomographic measurement, the number of projections was set to 1200 during a sample rotation of 180°. At each projection, a 5-step fringe scan method was used to calculate a differential phase image. Then, the differential phase image was integrated along the horizontal direction to generate a phase image. A phase tomogram was reconstructed from 1200 phase images using the conventional filtered back projection method.

### Estimation of the mass (tissue) density

The mass density *ρ* (g/cm^3^) is proportional to the phase factor *δ* in the X-ray refractive index, as shown in the following equation:

1 $$\delta =\frac{{\lambda }^{2}{r}_{e}{N}_{A}}{2\pi }\frac{Z}{M}\rho ,$$

where λ is the X-ray wavelength, *r*_e_ is the classical electron radius, *N*_*A*_ is Avogadro’s constant, and *Z/M* is the ratio of the number of electrons to the molecular weight of the sample. In most soft tissues, *Z/M* is assumed to be 0.55 (NIST: X-ray mass attenuation coefficients—Table 2. https://physics.nist.gov/PhysRefData/XrayMassCoef/tab2.html). The difference in the X-ray refractive index *Δδ* between the sample and water can be obtained from X-ray phase tomography^82^. Therefore, the difference in the density *Δρ* between the sample and water can also be estimated using Eq. (1). Finally, the mass density ρ of the sample is estimated from *Δρ* + *ρ*_*water*_ (= 1.0 g/cm^3^).

### 3D modeling of the liver and intrahepatic vessels and quantitative data processing

The computed tomography data from each mouse fetus were uploaded to advanced 3D image processing and quantification software (Amira, version 5.4.3, Thermo Fisher Scientific, MA. https://www.thermofisher.com), and extraction was performed using color contrast. The sample sizes were guided by a previous publication using synchrotron X-ray^83^. Liver and hepatic vessels were extracted from all mouse fetuses. The volume of the extracted liver was derived by forming multiple tetrahedrons with the center point as the apex in Amira. Intrahepatic blood vessels were extracted from the umbilical vein just before they entered the liver and branched. The blood flow rate was determined by deriving the vessel diameter, which is proportional to the multiplier of the vessel diameter according to Murray’s law^47,84^. For this purpose, we used an open-source toolkit (Vascular Modelling Toolkit, version 1.4.0, VMTK, www.vmtk.org) to derive the vessel diameter semiautomatically. The liver vessel tree geometries were clipped at the side branches such that the main vessel remained to reduce the computational cost (Supplementary Fig. 5C). The images were created using 3D image software (Amira).

### Statistics

The sample sizes for the animal studies were guided by a previous publication^85^. Values obtained from MPHs are presented as the means ± standard deviations (SD), and they are representative of at least three independent experiments. All measured variables from the synchrotron radiation micro-CT imaging were log(e) transformed for all statistical analyses. The significance of the differences between groups was determined using unpaired Student’s *t test*s and one-way repeated-measures ANOVA with Bonferroni’s post hoc tests for multiple comparisons as needed. Welch’s corrections were used when the variances between groups were unequal. Fisher’s exact test was used for categorical data. Nonparametric analyses of histological scores were conducted using a Kruskal–Wallis test with the unpaired, nonparametric Mann–Whitney *U* test as a post hoc analysis. *p* values < 0.05 were considered significant. Pearson’s correlation analysis was performed to analyze the association between two variables using GraphPad Prism ver. 6.0 software (GraphPad, San Diego, CA. https://www.graphpad.com). The other statistical analyses described above were performed using SPSS ver. 24 (SPSS, Chicago, IL. https://www.ibm.com/products/spss-statistics).

## Supplementary Information

Supplementary Information 1.

Supplementary Information 2.
