# Supplementary material for: A maternal high-fat diet induces fetal origins of NASH-HCC in mice
Source: Sci Rep. 2022 Jul 30;12:13136. doi: 10.1038/s41598-022-17501-8 (PMC9338981; doi:10.1038/s41598-022-17501-8)

**Supplementary Data**

**A maternal high-fat diet induces fetal origins of NASH-HCC in mice**

Takao Takiyama^1^, Toshihiro Sera^2^, Masanori Nakamura^3^, Masato Hoshino^4^, Kentaro Uesugi^4^, Shin-ichi Horike^5^, Makiko Meguro-Horike^5^, Ryoichi Bessho^1^, Yuri Takiyama^1^, Hiroya Kitsunai^1^, Yasutaka Takeda^1^, Kazuki Sawamoto^1^, Naoto Yagi^4^, Yuji Nishikawa^6^, Yumi Takiyama^1*^

^1^Division of Diabetes, Department of Medicine, Asahikawa Medical University, Japan.

^2^Department of Mechanical Engineering, Faculty of Engineering, Kyushu University, Japan.

^3^Department of Electrical and Mechanical Engineering, Nagoya Institute of Technology, Japan.

^4^Research & Utilization Division, Japan Synchrotron Radiation Research Institute, Japan.

^5^Advanced Science Research Center, Kanazawa University, Kanazawa, Ishikawa 920-0934, Japan.

^6^ Department of Pathology, Asahikawa Medical University.

**^*^** Corresponding author: Yumi Takiyama, MD, PhD.

Division of Diabetes, Department of Medicine, Asahikawa Medical University,

2-1-1-1 Midorigaoka higashi, Asahikawa 078-8510, Japan.

E-mail address: taka0716@asahikawa-med.ac.jp

Phone number: 81-166-68-2454, fax number: 81-166-68-2459

**Supplementary Table 1. Laboratory data of offspring from CD- and HFD-fed dams at 15 weeks of age.**

| Offspring | |  | FBS (mg/dl) | HbA1c  (mmol/  mol) | HbA1c  (%) | ALT  (IU/l) | NEFA  (μEq/l) | BW  (g) | SBP  (mmHg) | Food (g/day) |  |
| --- | --- | --- | --- | --- | --- | --- | --- | --- | --- | --- | --- |
| CD | Mean | | 74.0 | 15.52 | 3.6 | 17.0 | 608.0 | 21.98 | 100.0 | 2.33 | |
| n=5~7 | SD | | 17.0 | 4.84 | 0.44 | 4.20 | 66.61 | 0.90 | 11.54 | 0.97 | |
| HFD | Mean | | 52.9 | 8.65 | 2.9 | 27.7 | 911.0 | 22.55 | 98.2 | 3.45 | |
| n=7 | SD | | 8.71 | 3.67 | 0.34 | 5.96 | 242.38 | 1.82 | 7.69 | 0.54 | |
| P-value |  | | 0.012 | 0.011 | 0.011 | 0.002 | 0.016 | 0.476 | 0.735 | 0.027 | |

BW: body weight (g), FBS: fasting blood glucose (mg/dl), HbA1c: hemoglobin A1c (IFCC: mmol/mol, NGSP; %), ALT: alanine aminotransferase (IU/l), NEFA: nonesterified fatty acids (μEq/l), SBP: systolic blood pressure (mmHg), food: food intake (g/day). Values shown represent the mean and standard deviation (SD). The significance of the differences between groups was determined by unpaired Student’s *t*-tests. Welch’s corrections were used when the variances between groups were unequal.

**Supplementary Table 2. HORMAD1 associated cancers.**

| Reference | Cancer | Samples | Observations |
| --- | --- | --- | --- |
| Aung *et al* ^27^ | Gastric cancer | Gastric cancer specimens | HORMAD1 was over expressed in >45% of gastric cancers |
| Watkins *et al.* ^28^ | Triple Negative Breast Cancer (TNBC) | 142 frozen microdissected primary invasive TNBCs Breast cancer cell lines | *HORMAD1* as a driver homologous recombination deficiency in TNBC |
| Zong *et al*.^29^ | Triple Negative Breast Cancer | 640 TNBC samples Breast cancer cell lines | HORMAD1 promotes docetaxel resistance in triple negative breast cancer by enhancing DNA damage tolerance |
| Nichols *et al.*^30^ | Lung cancer | Non-Small Cell Lung Cancer cell lines Xenograft experiments | HORMAD1 specifies resistance to oxidative stress |
| Liu *et al.*^31^ | Lung cancer | 91 Lung adenocarcinoma tissues Lung cancer cell lines Xenograft experiments | HORMAD1 mediates EMT to promote tumor growth and metastasis |
| Liu *et al*.^32^ | Ovarian cancer | Ovarian cancer cell lines | HORMAD1 disrupts nuclear localization of MCM8-MCM9 complex and compromises DNA mismatch repair. |
| Shahzad *et al.*^33^ | Ovarian cancer | Ovarian cancer cell lines 90 frozen samples from patients | *HORMAD1* siRNAs results in significantly reduced VEGF protein levels and microvessel density |

| **Supplementary Table 3: Catalog numbers and sequences of siRNAs used in this study.** |
| --- |

|  |  |
| --- | --- |
|  | |
|  | |
|  | |

The negative control was a pool of four siRNAs designed for minimal targeting of mouse genes (ON-TARGETplus nontargeting control pool). All siRNAs were Dharmacon ON-TARGETplus siRNAs (Horizon Discovery, a PerkinElmer company, Cambridge, GB).

**Supplementary Figure legends**

**Supplementary Figure 1.** Maternal high-fat diet (HFD) does not affect the weight gain in offspring. Weight was measured from weaning at 4 weeks to 14 weeks in male offspring of CD-fed dams (CD) or HFD-fed dams (HFD). The significance of differences between groups was determined using two-way repeated-measures ANOVA with Bonferroni’s multiple comparison post hoc tests. *P*-values <0.05 were considered significant. There is no interaction between the groups and the time (*p*>0.05). The body weight of offspring from the HFD-fed dams dose not differed from that of offspring from CD-fed dams (p>0.05).

**Supplementary Figure 2.** A maternal high-fat diet (HFD) causes NASH in C57BL/6N offspring at 15 weeks of age. Hematoxylin and eosin staining showing microvesicular hepatic steatosis in the livers of 15-week-old C57BL/6N male offspring from HFD-fed dams (top panel). **(A)** Atypical cells accompanied by oval cell proliferation. **(B)** Large and thick hepatic arteries. **(C)** Atypical cells accompanied by large nuclei and eosinophilic cytoplasm in the livers of 15-week-old C57BL/6N female offspring from HFD-fed dams. **(D)** Large and thick hepatic arteries. Scale bars in the low magnification images: 300 μm. Scale bars in the high magnification images (A, B, C): 30 μm.

**Supplementary Figure 3.** Maternal HFD induced liver lesions independent of sex.

There is no difference in the occurrence of liver lesions between male or female offspring from CD- or HFD-fed dams in C57BL/6J. Fisher’s exact test was used for comparisons of male or female groups. *P*-values <0.05 were considered significant.　CD; 16 male offspring and 14 female offspring from 4 dams, HFD; 15 male offspring and 13 female offspring from 4 dams.

**Supplementary Figure 4.** Oval cell proliferation in hepatic tumors of offspring from HFD-fed dams. **(A)** Hematoxylin and eosin staining showing microvesicular hepatic steatosis and neoplastic tumors in the livers of 33-week-old C57BL/6J mice fed a HFD. The two panels below show magnified images of the sections identified by the squares in the top (solid line) and middle (dotted line) panels. **(B)** Many oval cells adjacent to the tumor **(**blue arrows). **(C)** High-power view of the area of oval cell proliferation in the liver. Scale bars in the top and middle panels: 300 μm. Scale bars in the lower panel: 30 μm.

**Supplementary Figure 5.** Synchrotron radiation micro-CT at Spring-8 (Hyogo, Japan). **(A)** The vascular structure of liver tissue samples was visualized using contrast medium and the synchrotron micro-CT system in BL20B2 at SPring-8. For each sample, 1800 radiographic images were acquired. The format of the 3D image was 2048 × 2048 × 1320 pixels. 1 pixel=15.5 μm. **(B)** X-ray phase tomography using a grating interferometer. A phase tomogram was reconstructed from 1200 phase images. The field of view was 14.2 mm in width. 1 pixel=3.47 μm. **(C)** 3D modeling of the intrahepatic vessels. 3-D image processing and quantification was achieved with Amira software (Thermo Fisher Scientific, MA. version 5.4.3, https://www.thermofisher.com), and extraction was done using color contrast. Intrahepatic blood vessels were extracted from the umbilical vein just before it entered the liver and branched. IVC: inferior vena cava, LPV: left portal vein, LHV: left hepatic vein, DV: ductus venosus, RHV: right hepatic vein, RPV: right portal vein, UV: umbilical vein.

**Supplementary Figure 1.**


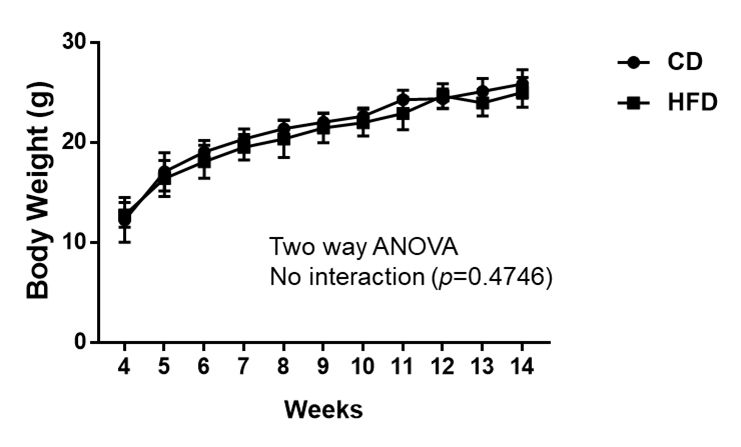


**Supplementary Figure 2.**


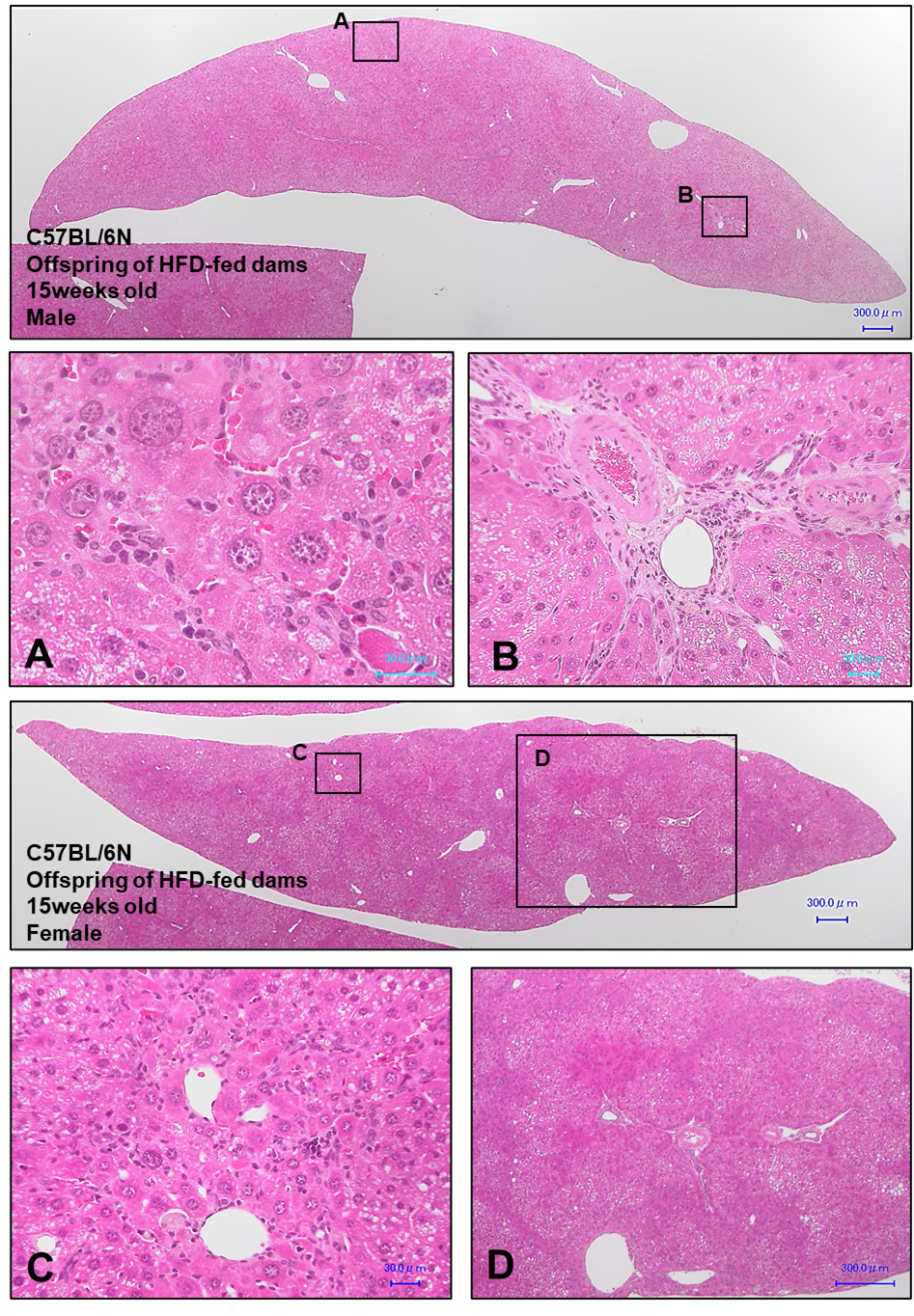


**Supplementary Figure 3.**

**
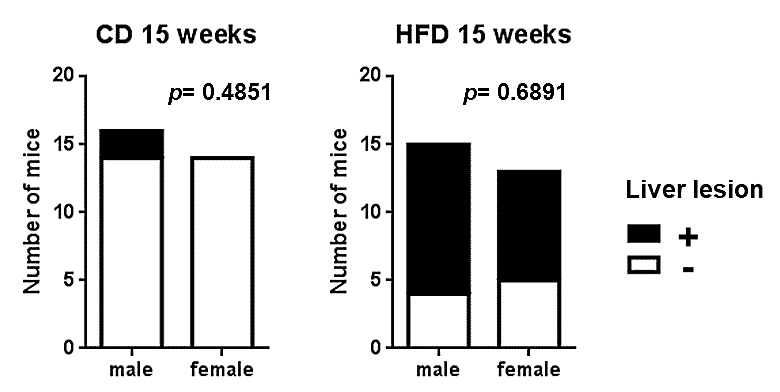
**

**
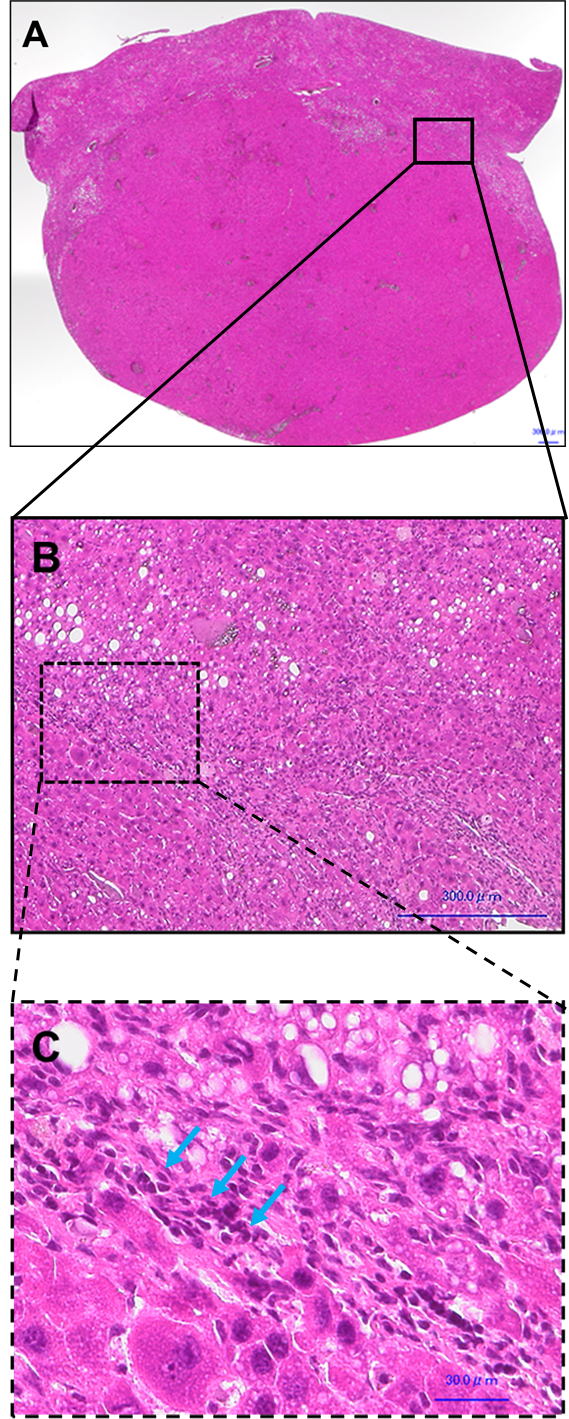
Supplementary Figure 4.**

**Supplementary Figure 5.**


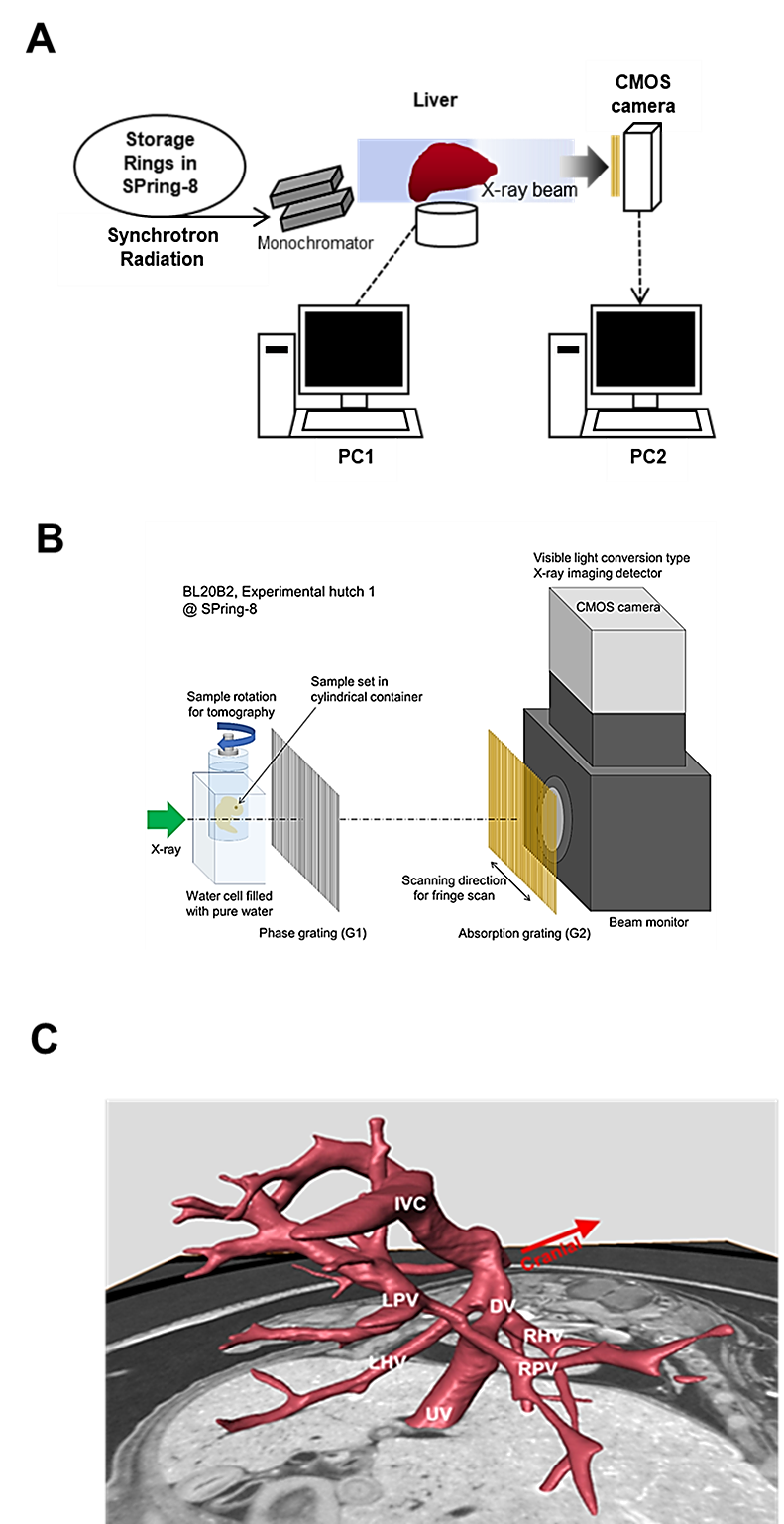

Supplement: Supplementary file 1 — Supplementary Information 1. [file 41598_2022_17501_MOESM1_ESM.docx]
