# Supplementary material for: A maternal high-fat diet induces fetal origins of NASH-HCC in mice
Source: Sci Rep. 2022 Jul 30;12:13136. doi: 10.1038/s41598-022-17501-8 (PMC9338981; doi:10.1038/s41598-022-17501-8)

Figure legend. Uncropped images for which boxed areas are shown in the panels B and D of Figure 3 and panels D of Figure 4. **(a)** HIF-1α, HORMAD1 and β-actin were evaluated by immunoblot analysis of livers at 14.5dpc from 3 fetuses of CD-fed dams and 5 fetuses of HFD-dams. **(b)** HIF-1α, HORMAD1 and β-actin were evaluated by immunoblot analysis of livers of 15 weeks old C57BL/6J male mice from 4 mice/group. **(c)** HIF-1α, HORMAD1 and β-actin were evaluated by immunoblot analysis of mouse primary hepatocytes under normoxic or hypoxic conditions. The samples (standardized to 40 μg of total cell lysates per lane) were analyzed on 4-12 %Tris-Glycine gel (Invitrogen) using HiMark™ Pre-stained protein standard (Invitrogen). After developing the HIF-1α, the blot was stripped and re-probed for HORMAD1 and/or β-actin. To confirm the total protein level, after immunodetection, membrane was stained by Coomassie brilliant blue solution (CBB) . The total protein level was consistent in all samples.

a. HIF-1α, HORMAD1 and β-actin of Figure 3B

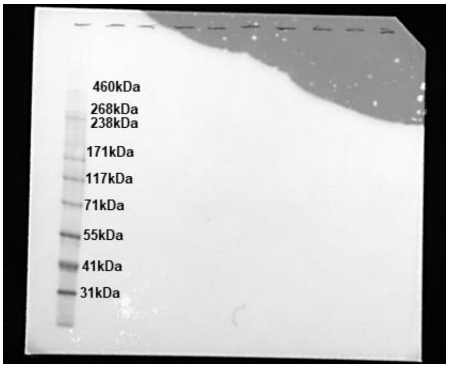

HIF-1α

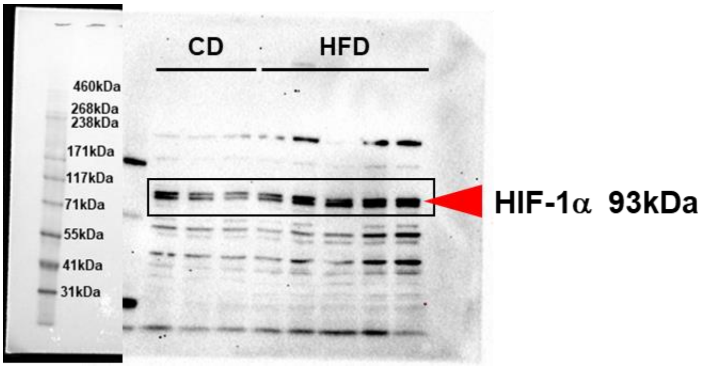

HORMAD1

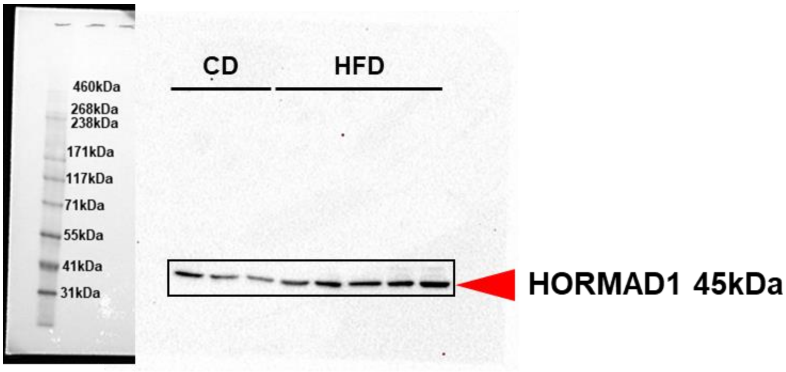

β-actin

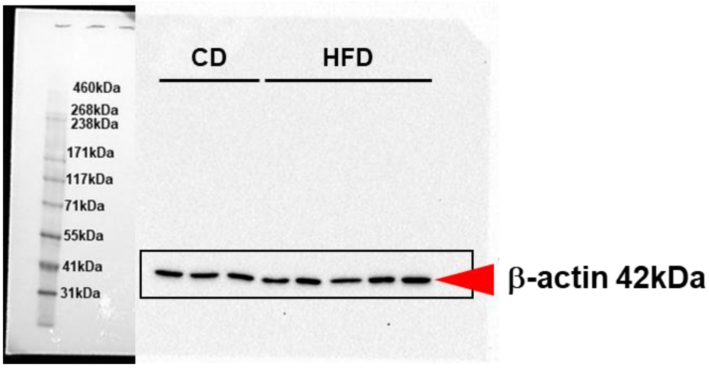

b. HIF-1α, HORMAD1 and β-actin of Figure 3D

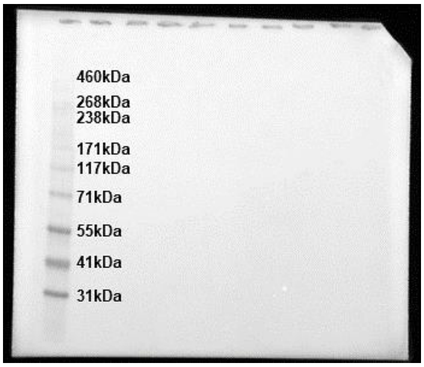

HIF-1α

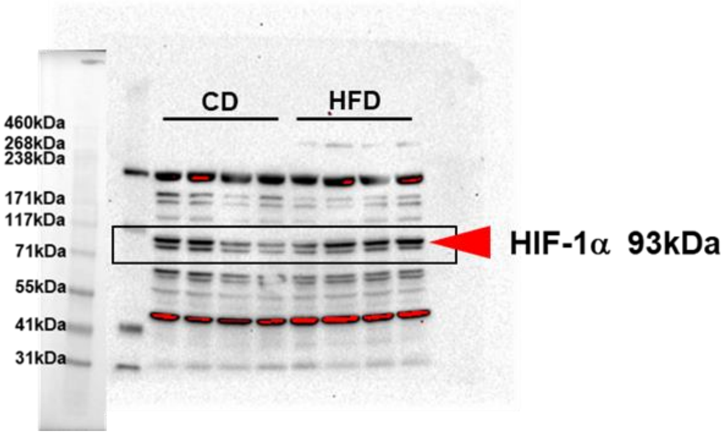

HORMAD1

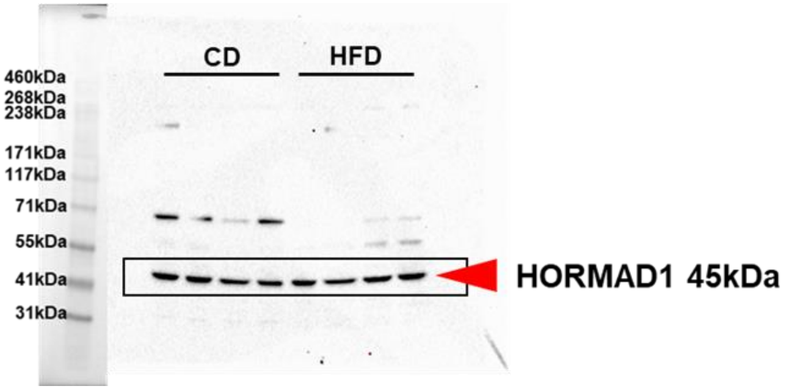

β-actin

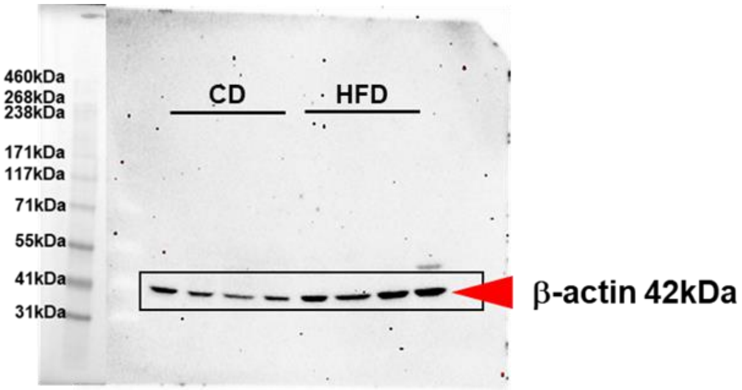

c. HIF-1α, HORMAD1 and β-actin of Figure 4D

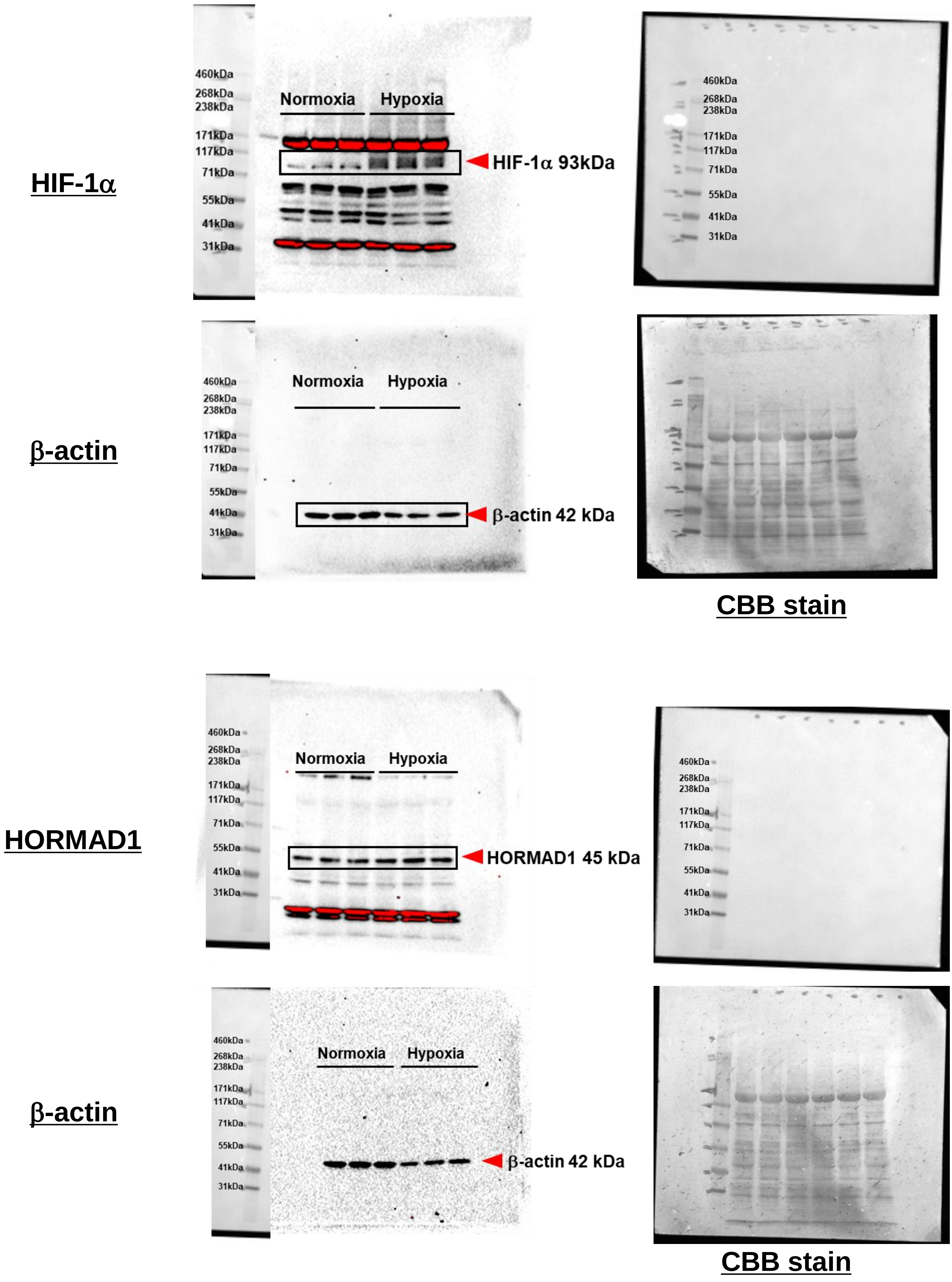

Supplement: Supplementary file 2 — Supplementary Information 2. [file 41598_2022_17501_MOESM2_ESM.pdf]
